# Systematic Review and Network Meta-analysis of Acupuncture Combined with Massage in Treating Knee Osteoarthritis

**DOI:** 10.1155/2022/4048550

**Published:** 2022-08-13

**Authors:** Zhen Wang, Yongquan Wang, Congan Wang, Xujie Li, Ziyang Zhou, Lijuan Zhang, Miaoxiu Li, Yankun Pan, Tiyong Jiao, Xiaoyun Shi, Qing Liu

**Affiliations:** ^1^Shandong University of Traditional Chinese Medicine, Jinan, China; ^2^Affiliated Hospital of Shandong University of Traditional Chinese Medicine, Jinan, China; ^3^Neck, Shoulder, Waist and Leg Pain Hospital Affiliated to Shandong First Medical University, Jinan, China

## Abstract

**Background:**

Knee osteoarthritis is a common clinical disease with frequent occurrence. More and more studies have shown that external therapies such as acupuncture and massage are beneficial to the treatment of knee osteoarthritis.

**Objective:**

The purpose of this systematic review and meta-analysis of randomized controlled trials (RCTS) was to evaluate the efficacy and safety of acupuncture and massage combined with treatment of KOA and to provide some reference for clinical treatment of KOA.

**Methods:**

Network meta-analysis was used to evaluate the efficacy of acupuncture combined with massage in the treatment of knee osteoarthritis. PubMed, Cochrane Library, Web of Science, Embase, Chinese Knowledge Infrastructure (CNKI), Chinese Biomedical Literature Database (CBM), VIP, and Wanfang were searched by computer for randomized controlled trials on acupuncture combined with massage in the treatment of knee osteoarthritis. All researchers independently screened the literature, extracted data, and evaluated quality, and studies that met the quality criteria were analyzed using Stata16.0 software.

**Results:**

A total of 3076 articles were retrieved, and finally, 49 studies involving 10 acupuncture combined with massage methods were included. The total sample size was 4458, including 2182 in the experimental group and 2276 in the control group. The results of network meta-analysis showed the following: in terms of effective rate, the optimal first three interventions were floating needle+massage, needle knife+massage, and silver needle+massage; in terms of reducing VAS score, the optimal first three interventions were common acupuncture+massage, needle knife+massage, and warm needle+massage; in terms of improving total Lysholm index score, the optimal first three interventions were silver needle+massage, electroacupuncture+massage, and needle knife+massage; in terms of reducing total WOMAC score, the optimal first three interventions were silver needle+massage, electrothermal needle+massage, and common acupuncture+massage; in terms of reducing WOMAC stiffness score, the optimal first three interventions were warm needle+massage, silver needle+massage, and common acupuncture+massage; and in terms of reducing WOMAC joint function score, the optimal first three interventions were silver needle+massage, warm needle+massage, and common acupuncture+massage.

**Conclusion:**

The results showed that acupuncture combined with massage could improve the clinical therapeutic effect of patients with knee osteoarthritis. Limited by the quality of the included studies, the conclusions obtained still need to be further validated.

## 1. Introduction

Knee osteoarthritis (KOA) is one of the most prevalent forms of this degenerative joint disease and is associated with pain, functional impairment and a high economic cost [1]. Knee osteoarthritis is good in the elderly, and the incidence is as high as 30% to 50% in people over 65 years of age [2]. The Osteoarthritis Research Society International (OARSI) recommends that the first-line management of knee osteoarthritis is still conservative treatment rather than surgery, which emphasizes the importance of conservative treatment in the diagnosis and treatment of knee osteoarthritis [3]. Drug treatment is commonly used in clinical practice, mainly including analgesics, nonsteroidal anti-inflammatory drugs, and corticosteroid injection. Although the above drugs have certain efficacy, they also have greater side effects [4, 5]. In recent years, a large number of clinical studies and applications have shown that acupuncture and massage can remarkably improve symptoms and motor function of knee joints for knee osteoarthritis patients, which has obvious efficacy, rapid effect, and no obvious adverse reactions [6, 7]. However, the advantages and disadvantages between different acupuncture and massage therapy methods are not clear; therefore, it is necessary and practical to evaluate the clinical efficacy of different acupuncture and massage therapy for KOA. Several traditional and network meta-analysis have proved that acupuncture and massage treatment of knee osteoarthritis do have advantages [8–10], but more for acupuncture and massage comparison and contrast between different acupuncture and western medicine, not to compare a variety of acupuncture therapy in combination with the massage, although some network meta-analysis involving acupuncture combined massage intervention measures, but it is not only limited to acupuncture and massage; Chinese and Western medicines are often added for internal and external use [11, 12]. In this study, the network meta-analysis method was used for the first time to compare the effectiveness of different acupuncture combined with massage, so as to provide evidence-based medical basis for clinical selection of the best program. It is planned to use network meta-analysis method to evaluate the efficacy of commonly used acupuncture and massage in the prevention and treatment of KOA and to provide evidence-based basis for the clinical selection of acupuncture and massage in the prevention and treatment of KOA.

## 2. Materials and Methods

### 2.1. Inclusion Criteria

#### 2.1.1. Study Types

In a published randomized controlled trial (RCT), the language is limited to Chinese and English.

#### 2.1.2. Study Subjects

For patients with knee osteoarthritis, the diagnostic criteria refer to the Guidelines for the Diagnosis and Treatment of Osteoarthritis [13–15], Jishuitan Practical Orthopaedics [16], Practical Arthritis Diagnosis and Therapy [17], the criteria established by the State Administration of Traditional Chinese Medicine [18], and the criteria established by the American Rheumatism Association [19, 20], which are not limited by the patient's age, gender, and nationality.

#### 2.1.3. Interventions

Acupuncture combined with massage was used in the experimental group, and acupuncture therapy included electrothermal needle, floating needle, fire needle, silver needle, beryllium needle, electroacupuncture, warm needle, needle knife, common acupuncture, and catgut embedding needle knife. The control group was treated with massage alone, above-mentioned acupuncture therapy alone, or above-mentioned acupuncture combined with massage. Note that massage-related techniques of different names are collectively referred to as massage to evaluate, including Chinese tuina, loosening, Maitland, joint mobilization, and self-created massage. A brief introduction of the different acupuncture and massage treatments is provided in supplementary Table S17.

#### 2.1.4. Outcome Measures

The primary outcome measure was the clinical effective rate, the follow-up visual analogue scale (VAS), Lysholm index total score, and WOMAC osteoarthritis index total score; the secondary outcome measures were WOMAC stiffness score, WOMAC joint function score, and the occurrence of adverse reactions.

### 2.2. Exclusion Criteria

The following are the exclusion criteria: animal experiment, inconsistent intervention measures, repeatedly published literatures, random grouping not mentioned, diagnosis or efficacy evaluation criteria not mentioned, obvious statistical error in the literatures, the study which cannot be obtained in full text, the study without the above outcome indicators, and combined with other diseases.

### 2.3. Literature Search Strategy

A computerized search of published relevant studies on acupuncture combined with massage in the treatment of KOA was performed. PubMed, Cochrane Library, Web of Science, Embase, China National Knowledge Infrastructure (CNKI), Chinese Biomedical Literature Database (CBM), VIP, and Wanfang databases were searched. Chinese search terms included xi gu guan jie yan, xi guan jie gu xing guan jie yan, xi guan jie gu guan jie bing, xi guan jie tui hang xing guan jie yan, xi bi, xi tong zheng, zhen jiu, dian zhen, wen zhen, zhen ci, dong jin zhen, huo zhen, zhen dao, ren zhen, ci luo fang xue, fu zhen, dian re zhen, yin zhi zhen, pi zhen, mai xian zhen dao, zhen, tui na, song dong shu, shou fa, li jin and an mo. English search terms included Knee osteoarthritis, Osteoarthritis, Knee, Osteoarthritis of Knee, Acupuncture, Electroacupuncture, warm acupuncture, warm needling, Moving tendon needle, fire needle, Acupotomy, Blade Needle, Bloodletting acupuncture, Floating needle, Electrico therapy, Silver needle, Stiletto needle, needle, Tuina, Massage, Zone Therapy, needle, Joint mobilization, etc. The search was performed using a combination of subject headings and free words from database establishment to July 15, 2021, and the search was not limited by the type of publication. Supplementary Table S1 lists the search strategy for the respective database.

### 2.4. Literature Screening and Data Extraction

Two investigators independently screened the literatures, extracted the data, and cross-checked according to the predetermined screening criteria. In case of any difference, it could be decided by discussion or a third party. After checking the imported literatures with endnote literature management software, the primary screening was performed by reading the title and abstract of literatures. The literatures that obviously did not meet the inclusion criteria were excluded. The full text of literatures that may meet the inclusion criteria was further read and rescreened to determine whether they were finally included. If needed, the authors of the original study can be contacted by telephone or mail to obtain information that is very important for this study. Excel data extraction form was established to extract the data, mainly including the first author of the included literature, publication year, sample size, age and course of disease, intervention measures, outcome indicators, and course of treatment.

### 2.5. Evaluation of Risk of Bias of Included Studies

The quality evaluation of literatures was evaluated by 2 investigators according to the risk of bias assessment tool in Cochrane Reviewers Handbook 6.1.0 [21], mainly including the following 7 aspects: random sequence generation; allocation concealment; implementation of blind method for patients and trial personnel; implementation of blind method for outcome assessors; incomplete result data; selective reporting; and other biases (such as potential bias related to special study design in studies, and false statement). Eventually, it is necessary to make a judgment on “low risk,” “high risk,” and “unclear risk” in the literatures.

### 2.6. Statistical Analysis

The statistical method for network meta-analysis is based on a frequency framework, and all outcome measures are analyzed using a random-effects model for data analysis. If the evaluation indicators of this study are continuous variables, standardized mean difference (SMD) is used as the effect size; if they are binary variables, odds ratio (OR) is used as the effect size, and the corresponding 95% confidence interval (CI) is calculated [22]; Stata16.0 software is used to select the frequentist framework random effects model for network meta-analysis, ranking group command is used for data preprocessing, Network relationship diagram is drawn for the comparison between the interventions of each outcome indicator, efficacy ranking is performed, and cumulative probability ranking diagram is drawn to obtain the area under the curve (SUCRA). In the reticulation diagram, the dot area represents the number of patients with relevant interventions, and the thickness of the line between points represents the number of included studies [23]. SUCRA is expressed as a percentage. When SUCRA is 100%, it indicates that the intervention is absolutely effective, while when it is 0, it indicates that the intervention is absolutely ineffective [24, 25]. The inconsistency test is mainly used to assess the degree of consistency between the direct comparison results and the indirect comparison results. When there is closure ring, it is necessary to make a test for inconsistency. Finally, the presence of small sample effects in the network was identified by drawing a funnel plot. Review Manager 5.4 software will be used for literature quality evaluation in this study.

## 3. Results

### 3.1. Literature Search

**Figure 1:**
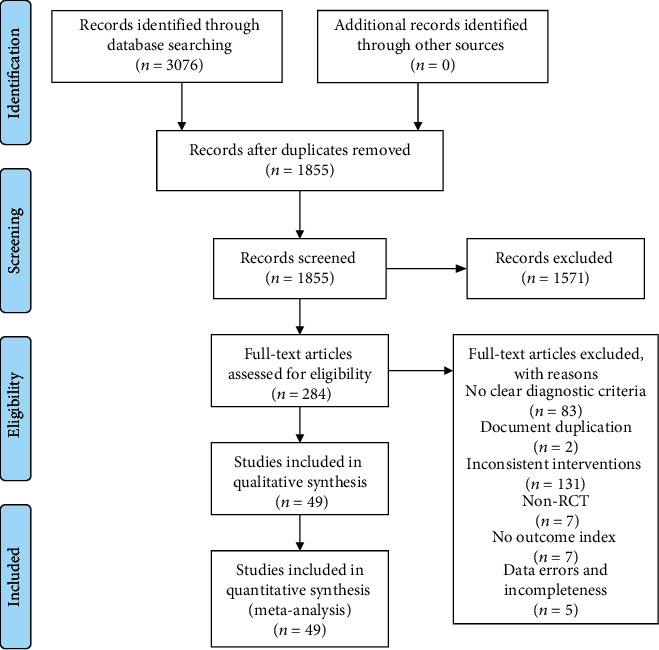
Literature search process.

A total of 3076 literatures were retrieved, among which 49 [26–74] were included after layer-by-layer screening, of which 2 [73, 74] were 3-arm trials and the rest 47 were double-arm trials.  Figure 1 is illustrated for the literature retrieval process. A total of 4458 patients were included, all of whom had a definite diagnosis of knee osteoarthritis, including 2182 in the test group and 2276 in the control group. Basic characteristics of the included studies are shown in Table 1.

### 3.2. Quality Evaluation of Included Literatures

**Figure 2:**
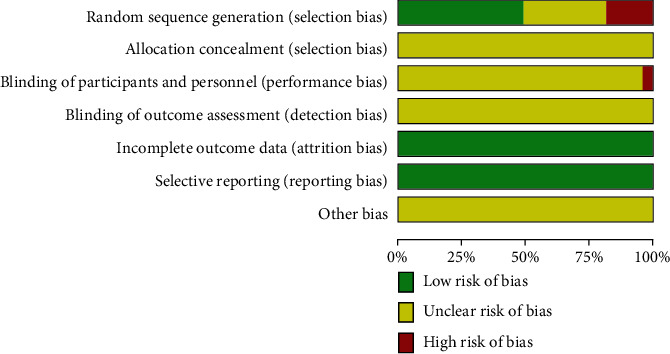
Literature quality evaluation results.

All 49 included studies were Chinese RCTs, and the baseline data of the control group were comparable. Twenty-four studies reported the generation method of specific random sequences, of which 22 studies used random number table for random allocation [26, 27, 29–32, 36, 38, 42, 44–46, 51, 53, 55, 58, 60, 62, 64, 72–74], 2 studies used lottery for random allocation [68, 71], rated as “low risk,” 7 studies used order of presentation for allocation [41, 47, 49, 65, 66, 69, 70], 1 study used voluntary patient allocation [37], 1 study used different treatment methods [33], and 1 study used doctor's wishes for allocation [61] rated as “high risk”; the remaining 15 studies mentioned randomization but did not specify the specific method, rated as “unclear risk.” Two studies mentioned the implementation of the blind method, and both performed single-blinding [53, 55] rated it as “high risk”; the remaining 47 studies did not report the use of the blind method and rated it as “unclear risk.” All 49 studies did not describe allocation concealment and were rated as “unclear risk.” None of the 49 studies mentioned the implementation of blind method for result evaluation and were rated as “unclear risk.” All 49 studies reported the outcome measures that were expected to be measured. No premature termination of the trial was found in the study. The incomplete data and selective reporting were rated as “low risk.” None of the 49 studies described other biases in detail and were rated as “unclear risk.” The results of literature quality evaluation are shown in Figure 2. The results of risk of bias summary are shown in Supplementary Material Figure S1.

### 3.3. Clinical Effective Rate

#### 3.3.1. Evidence Network

**Figure 3:**
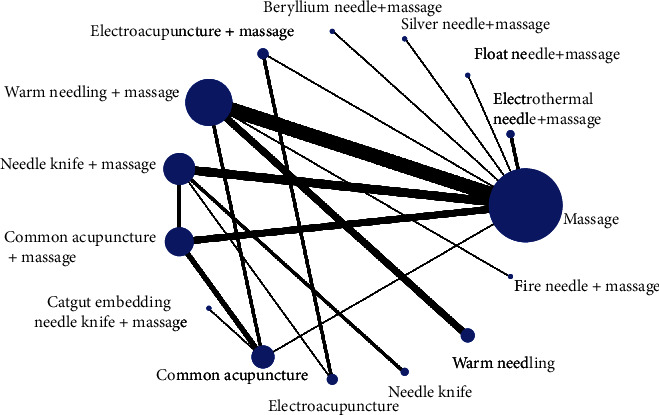
Network relationship diagram of clinical effective rate.

Forty RCTs reported clinical response rates [26–28, 30–33, 35–45, 47–49, 51–56, 58–59, 61–68, 70, 72, 74] , involving 15 treatments, the network relationship was generally centered on massage therapy, the dot size represented the sample size of this intervention, and the line thickness represented the number of RCTs using two-point therapy, which could be seen to contain three closed rings, as shown in Figure 3.

#### 3.3.2. Network Meta-analysis

Network meta-analysis of the included studies yielded 105 pairwise comparisons, combined with OR and 95% CI, network meta-analysis results showed that compared with common acupuncture+massage, warm needling+massage, fire needling+massage, catgut embedding needle knife+massage, needle knife, massage, warm needling, common acupuncture, and electroacupuncture, the intervention effect of floating needle+massage was better; compared with common acupuncture+massage, warm needling+massage, needle knife, massage, warm needling, common acupuncture, and electroacupuncture, needle knife+massage had better efficacy; compared with massage, warm needling, common acupuncture, and electroacupuncture, silver needle+massage, electrothermal needle+massage, and common acupuncture+massage had better efficacy; compared with massage, warm needling, and common acupuncture, warm needling+massage had better efficacy; electroacupuncture+massage had no statistical significance compared with electroacupuncture, as shown in Table 2.

#### 3.3.3. SUCRA Probability Ranking

**Figure 4:**
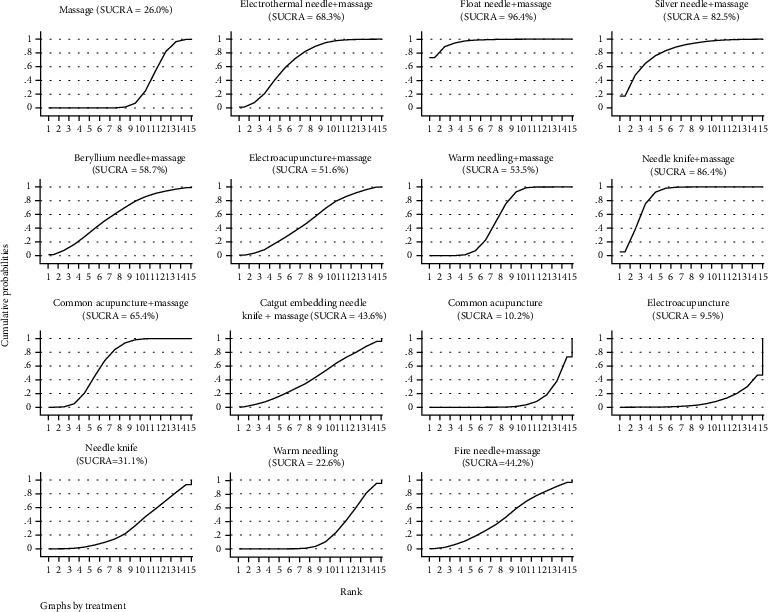
SUCRA of clinical effective rate.

According to SUCRA results, floating needle+massage may be the most effective intervention, and the results of SUCRA probability ranking from high to low are as follows: floating needle+massage>needle knife+massage>silver needle+massage>electrothermal needle+massage>common acupuncture+massage>beryllium needle+massage>warm needle+massage>electroacupuncture+massage>electroacupuncture+massage>fire needle+massage>catgut embedding needle knife+massage>needle knife>massage>warm needle>common acupuncture>electroacupuncture, as shown in Figure 4.

#### 3.3.4. Publication Bias

**Figure 5:**
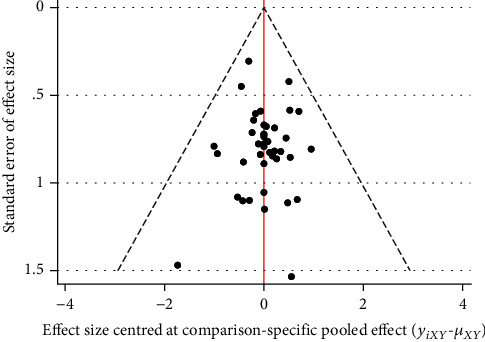
The funnel plot of clinical effective rate.

The funnel plot of publication bias showed poor symmetry, suggesting that there may be some publication bias, as shown in Figure 5.

### 3.4. Visual Analogue Scale

#### 3.4.1. Evidence Network

**Figure 6:**
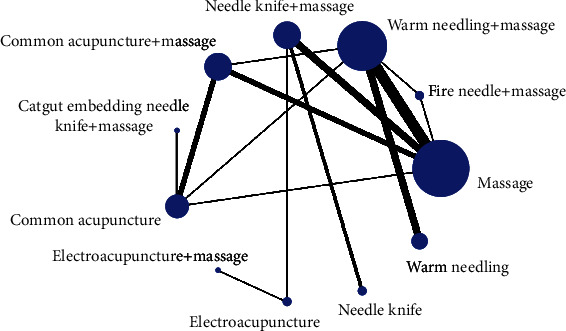
Network relationship diagram of visual analogue scale.

Twenty-eight RCTs reported VAS scores, involving 11 treatments, and the network relationship was generally centered on massage therapy, which can be seen to contain four closed rings, as shown in Figure 6.

#### 3.4.2. Network Meta-analysis

Network meta-analysis was performed on the included studies, yielding 55 pairwise comparisons.

Compared with massage, common acupuncture+massage (SMD = −1.67, 95% CI [-2.62, -0.72]), needle knife+massage (SMD = -1.59, 95% CI [-2.61, -0.57]), and warm needling+massage (SMD = −1.50, 95% CI [-2.19, -0.81]) had a better effect in reducing the total VAS score; compared with warm needling, common acupuncture+massage (SMD = −1.84, 95% CI [-3.25, -0.42]), needle knife+massage (SMD = −1.76, 95% CI [-3.31, -0.20]), and warm needling+massage (SMD = −1.66, 95% CI [-2.65, -0.68]) decreased the VAS score; compared with common acupuncture, common acupuncture+massage (SMD = −1.88, 95% CI [-2.93, -0.84]) and needle knife+massage (SMD = −1.80, 95% CI [-1.26, -0.71], 95% CI [-2.89, -0.53]) had better intervention effect; compared with needle knife, needle knife+massage (SMD = −1.86, 95% CI [-3.25, -0.48]) reduced VAS score, and other comparisons had no statistical difference, as shown in Table 3.

#### 3.4.3. SUCRA Probability Ranking

**Figure 7:**
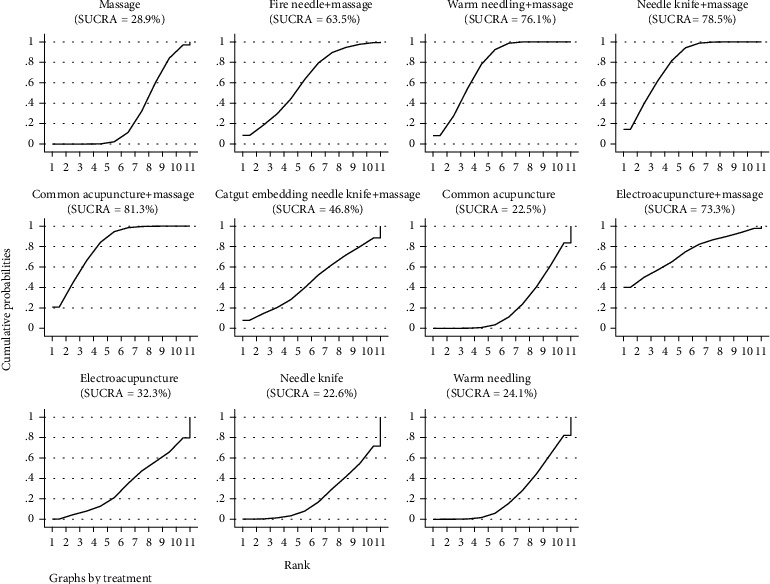
SUCRA of visual analogue scale.

According to the results of SUCRA, common acupuncture+massage may be the most effective intervention to reduce the total VAS score of patients, and the results of SUCRA probability ranking from high to low are as follows: common acupuncture+massage>needle knife+massage>warm needle+massage>electroacupuncture+massage>fire needle+massage>catgut embedding needle knife+massage>electroacupuncture>massage>warm needle>needle knife>common acupuncture, as shown in Figure 7.

#### 3.4.4. Publication Bias

**Figure 8:**
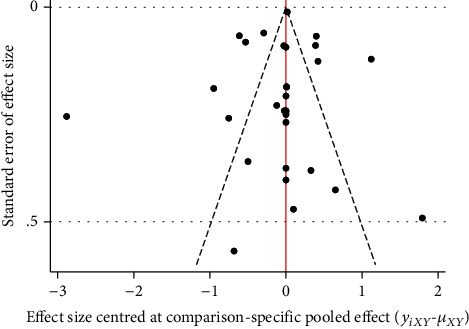
The funnel plot of visual analogue scale.

The funnel plot results showed poor symmetry, suggesting that there may be some publication bias, as shown in Figure 8.

### 3.5. Total Lysholm Score

#### 3.5.1. Evidence Network

**Figure 9:**
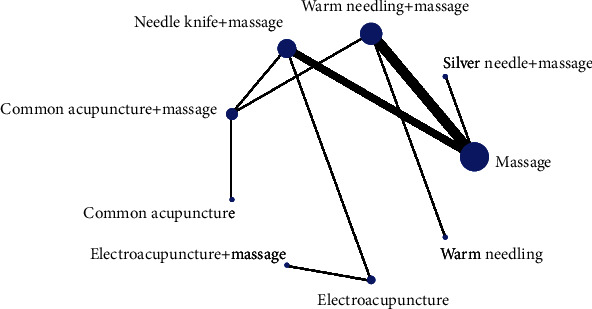
Network relationship diagram of total Lysholm score.

Fourteen RCTs reported total Lysholm score, involving nine treatments, as shown in Figure 9.

#### 3.5.2. Network Meta-analysis

Network meta-analysis was performed on the included studies, yielding 36 pairwise comparisons. Silver acupuncture+massage and electroacupuncture+massage was compared with the other 7 interventions reporting total Lysholm score improved the total Lysholm score, with better efficacy; in silver acupuncture+massage compared with electroacupuncture+massage (SMD = 2.63, 95% CI [-3.23, 8.50]), there was no statistical difference; in massage compared with electroacupuncture (SMD = 0.82, 95% CI [-1.24, 2.88]) and warm needling (SMD = 2.82, 95% CI [-0.36, 5.99]), there was no statistical difference; in electroacupuncture compared with warm needling (SMD = 1.99, 95% CI [-1.76, 5.74]), there was no statistical difference; other comparisons were statistically significant, as shown in Table 4.

#### 3.5.3. SUCRA Probability Ranking

**Figure 10:**
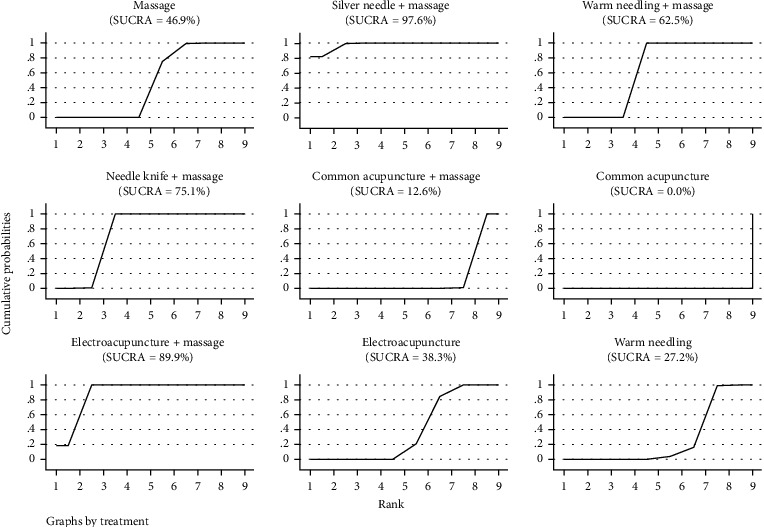
SUCRA of total Lysholm score.

According to the results of SUCRA, silver needle+massage may be the most effective intervention to reduce the total Lysholm score of patients, and the results of SUCRA probability ranking from high to low are as follows: silver needle+massage>electroacupuncture+massage>needle knife+massage>warm needle+massage>massage>electroacupuncture>warm needle>warm needle>common acupuncture+massage>common acupuncture, as shown in Figure 10.

#### 3.5.4. Publication Bias

**Figure 11:**
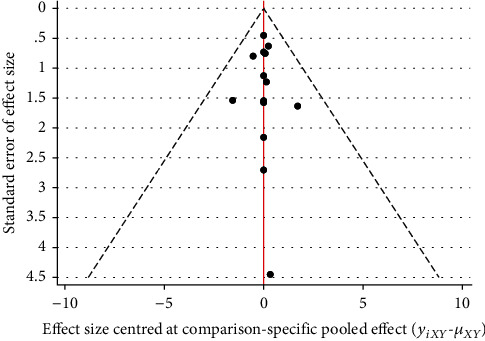
The funnel plot of total Lysholm score.

The funnel plot results showed poor symmetry, suggesting that there may be some publication bias, as shown in Figure 11.

### 3.6. Total WOMAC Score

#### 3.6.1. Evidence Network

Eight RCTs reported total WOMAC scores involving nine interventions. The results are shown in Supplementary Figure S2.

#### 3.6.2. Network Meta-analysis

Network meta-analysis was performed on the included studies, resulting in 36 pairwise comparisons and 29 comparisons with statistical differences. Compared with the other 8 interventions reporting total WOMAC score, silver needle+massage reduced total WOMAC score, with better efficacy; there was no significant difference between electric hot needle+massage and fire needle+massage (SMD = −0.62, 95% CI [-7.32, 6.08]) and common acupuncture+massage (SMD = −3.37, 95% CI [-6.96, 0.23]); there was no significant difference between common acupuncture+massage and fire needle+massage (SMD = −2.75, 95% CI [-9.09, 3.60]) and silver needle (SMD = −4.17, 95% CI [-10.31, 1.98]); there was no significant difference between fire needle+massage and silver needle (SMD = −1.42, 95% CI [-3.83, 0.99]); there was no significant difference between massage and warm needle+massage (SMD = −6.84, 95% CI [-15.68, 6.44]); there was no statistical difference; the other 29 comparisons had statistical difference. The results are shown in Supplementary Table S2.

#### 3.6.3. SUCRA Probability Ranking

According to the results of SUCRA, silver needle+massage may be the most effective intervention to reduce the total WOMAC score of patients, and the results of SUCRA probability ranking from high to low are as follows: silver needle+massage>electrothermal needle+massage>common acupuncture+massage>fire needle+massage>silver needle>massage>warm needle+massage>common acupuncture>warm needle. The results are shown in Supplementary Figure S3.

#### 3.6.4. Publication Bias

The funnel plot results showed poor symmetry, suggesting that there may be some publication bias. The results are shown in Supplementary Figure S4.

### 3.7. WOMAC Stiffness and Joint Function Scores

#### 3.7.1. Evidence Network

Six RCTs reported WOMAC stiffness and joint function scores involving seven interventions. The results are shown in Supplementary Figure S5.

#### 3.7.2. Network Meta-analysis

In terms of WOMAC stiffness score, compared with massage (SMD = −1.46, 95% CI [-2.52, -0.40]), common acupuncture (SMD = −2.84, 95% CI [-5.09, -0.59]) and warm needling+massage reduced WOMAC pain score, with better efficacy; silver needling+massage had better intervention effect compared with massage (SMD = −1.40, 95% CI [-2.79, -0.01]) and common acupuncture (SMD = −2.78, 95% CI [-5.20, -0.36]); compared with common acupuncture, common acupuncture+massage (SMD = −2.18, 95% CI [-3.62, -0.74]) had better effect in reducing WOMAC stiffness score, and other comparisons were not statistically different. The results are shown in Supplementary Table S3.

In terms of WOMAC joint function score, silver needle+massage had better efficacy in reducing WOMAC daily activity compared with the other six interventions reporting WOMAC joint function score; compared with the other six interventions reporting WOMAC joint function score, common acupuncture had the worst effect in reducing WOMAC joint function score; warm needle+massage had better intervention effect compared with common acupuncture+massage (SMD = −4.34, 95% CI [-7.79, -0.88]), massage (SMD = −7.34, 95% CI [-9.48, -5.19]), and silver needle (SMD = −7.44, 95% CI [-10.58, -4.29]); compared with massage (SMD = −3.00, 95% CI [-5.71, -0.29]), common acupuncture+massage could better reduce WOMAC joint function score, and there was no statistical difference in other comparisons, WOMAC joint function score. The results are shown in Supplementary Table S4.

#### 3.7.3. SUCRA Probability Ranking

In terms of WOMAC stiffness score, warm needling+massage may be the most effective intervention to reduce WOMAC pain score in patients, and the results of SUCRA probability ranking from high to low are as follows: warm needling+massage>silver needling+massage>common acupuncture+massage>warm needling>silver needling>massage>common acupuncture. The results are shown in Supplementary Figure S6.

In terms of WOMAC joint function score, silver needling+massage may be the most effective intervention to reduce WOMAC joint function score in patients, and the results of SUCRA probability ranking from high to low are as follows: silver needling+massage>warm needling+massage>common acupuncture+massage>warm needling>massage>silver needling>common acupuncture. The results are shown in Supplementary Figure S8.

#### 3.7.4. Publication Bias

In terms of WOMAC stiffness and joint function score, the funnel plot results showed poor symmetry, suggesting that there may be some publication bias. The results are shown in Supplementary Figure S7, 9.

### 3.8. Incidence of Adverse Reactions

Of the 49 articles, 9 reported the occurrence of adverse reactions. Among them, 7 had no adverse reaction [29, 36, 44, 45, 49, 51, 53]; 1 reported 4 cases of slight hemorrhage in the observation group [37]; 1 reported 3 cases of slight subcutaneous ecchymosis in the observation group, 2 cases of slight subcutaneous ecchymosis, and 1 case of slight subcutaneous hematoma in the silver needle group [73]. Overall, acupuncture combined with massage treatment of KOA showed only mild adverse effects and no serious adverse effects.

### 3.9. Sensitivity Analyses

To assess the robustness and reliability of results, we did two sensitivity analyses. First, the massage treatments included in this study can be divided into 3 types, of which 41 [28, 30–33, 35–37, 39–43, 45–50, 52–57, 59–74] are traditional Chinese tuina (the specific operations are carried out under the guidance of “Theory and Practice of Tuina”), 5 [26, 27, 34, 38, 44] are joint mobilization (including Maitland and Loosening, which are from European and American countries), and 3 [29, 51, 58] are self-created methods. Considering the potential heterogeneity that different massage methods may bring, but the latter two methods have less literature and could not be used for subgroup analysis, so sensitivity analysis was performed. We excluded 8 nonconventional massage studies [26, 27, 29, 34, 38, 44, 51, 58] to check the effect of nonconventional massage interventions on the results. Sensitivity analyses will be performed before and after exclusion. For the results of sensitivity analysis, only network meta-analysis of clinical effective rate, VAS score, and total Lysholm score can be performed.

In terms of clinical effective rate, 15 treatments for 3787 patients from 40 studies and 14 treatments for 3109 patients from 34 studies were included in the sensitivity analysis by total massage and conventional massage. Sensitivity analysis showed that the overall results were still robust, and the top three rankings did not change. Due to too few literatures included, the electrothermal needle+massage intervention measures in the conventional massage group failed to be retained, but this measure ranked lower and had little effect on the overall ranking. In terms of VAS score, 11 treatments for 2577 patients from 28 studies and 11 treatments for 2320 patients from 34 studies were included in the sensitivity analysis by total massage and conventional massage. Sensitivity analysis showed that needle knife+massage lost its ranking advantage over warm needling+massage, and the overall results were still robust. In terms of total Lysholm score, 9 treatments for 1454 patients from 14 studies and 9 treatments for 1319 patients from 12 studies were included in the sensitivity analysis by all massages and conventional massage; sensitivity analysis showed that the overall results remained robust. In conclusion, the overall results of the above indicators are robust, which may indicate that there may be no significant difference in the efficacy of different massage techniques in the treatment of knee osteoarthritis.

Secondly, we found that the methodological quality of some of the included studies was poor, which may lead to the occurrence of heterogeneity. Therefore, we removed the 12 literatures [33, 37, 41, 47, 49, 53, 55, 61, 65, 66, 69, 70] that were rated as high risk in the risk assessment and reconducted the network meta-analysis, compared with before elimination. As part of the indicators could not be operated after being removed, we only made network meta-analysis of major scores such as clinical effective rate, VAS score, and total Lysholm.

In terms of clinical effective rate, 11 articles [33, 37, 41, 47, 49, 53, 55, 61, 65, 66, 70] were excluded, and 15 interventions were used in both groups. The overall results were still robust, with the top three interventions unchanged and the bottom three interventions changed, but the overall ranking was not significantly affected. In terms of VAS score, 7 articles [33, 37, 55, 61, 65, 66, 69] were excluded, and 11 interventions were used in both groups. The overall results were still robust, and warm acupuncture+massage lost its ranking advantage over electroacupuncture+massage, which may be caused by improper use of methodology. In terms of total Lysholm score, two articles [37, 69] were excluded, and nine interventions were used in both groups. The overall results remained robust, with no change in all rankings. In conclusion, the overall results of the above indicators are stable, and the top two rankings remain unchanged, while some lower-ranked interventions change, indicating that heterogeneity may occur due to improper use of methodology, but the overall impact is not significant. Note that specific charts and data of sensitivity analyses are shown in pages 12-27 of Supplementary Figure S10-21 and Table S11-16.

### 3.10. Subgroup Analysis

In order to reduce the heterogeneity caused by the inconsistency of the treatment course, we divided the study into two subgroups of <3 weeks and ≥3 weeks for analysis. Regarding the outcomes in this analysis, only network meta-analysis of primary outcomes (clinical effective rate, VAS score, and total Lysholm score) can be performed.

In terms of total effective rate, in the <3-week subgroup, the results of the network meta-analysis and the SUCRA probability ranking are consistent with those before the grouping. Needle knife+massage showed superiority in the ≥3-week subgroup. The comparison between the <3-week and ≥3-week subgroups showed there was no significant difference between the two subgroups, except that the ranking of warming needle+massage was improved in the ≥3-week subgroup.

In terms of VAS score, in the <-week subgroup, the results of the network meta-analysis and the SUCRA probability ranking are consistent with those before the grouping. Needle knife+massage lost its ranking advantage over warm needling+massage in the ≥3-week subgroup. The comparison between the <3-week and ≥3-week subgroups showed there was no significant difference between the two subgroups, except that the ranking of warming needle+massage was improved in the ≥3-week subgroup. In terms of total Lysholm score, the comparison between the <3-week and ≥3-week subgroups showed common acupuncture+massage lost its ranking advantage over warm needling+massage in the ≥3-week subgroup and no significant difference in other comparisons.

This subgroup analysis shows that there was no significant difference between before and after grouping and between groups. This indicates the difference in the course of treatment has perhaps little effect on this study. However, due to the small amount of literature, some interventions are missing for some indicators after grouping, which may increase the heterogeneity of results. In the three indicators, the effect of warming needle+massage is better with the increase of the course of treatment, and the ranking is improved, which may be related to the slow onset of warming needle+massage. The results of subgroup analysis are shown in Supplementary Figure S22-27 and Table S11-16.

## 4. Discussion

Osteoarthritis of the knee affects an important part of the population, causing disability in many individuals and impairs a patient's quality of life significantly. The prevalence of KOA is expected to increase dramatically in the near future due to the increased life expectancy and an increasing rate of obesity of the world population [75, 76]. In recent years, complementary and alternative medicine (CAM) is being widely accepted and applied in clinical practice [77]. Acupuncture and massage therapy, as a supplementary alternative therapy, has a significant clinical effect in the treatment of knee osteoarthritis, without significant side effects [78, 79]. Traditional Chinese medicine classifies KOA as “bi zheng” (arthralgia syndrome). In China, acupuncture and massage therapies have been used in the treatment of arthralgia syndrome for thousands of years. Massage therapy can play a role in releasing adhesions, activating meridians, and regulating muscle function, and the main mechanism of action is to promote blood circulation and substance metabolism [80, 81]. Acupuncture can stimulate meridians and smooth meridians and qi and blood, and the main mechanism of action is to promote the release of central analgesic transmitters such as opioid peptides [82], both of which can effectively relieve the symptoms of KOA patients. The acupuncture therapy included in this paper can be generally divided into two types, one is to treat KOA by inhibiting the inflammatory response, releasing adhesions, stimulating the body's immune response and other mechanisms, such as common acupuncture, electroacupuncture, needle knife, floating needle, and beryllium needle; the other is to treat KOA by promoting blood circulation and dilating blood vessels through warming effects, such as silver needle, electrothermal needle, warm needle, and fire needle [83, 84]. At present, the clinical research of acupuncture and massage is increasing, there are many clinical acupuncture and massage programs for patients with KOA, in which the acupuncture and massage method has the best effect, acupuncture and massage alone or in combination; there is still no optimal treatment program. In this study, Network meta-analysis was used for the first time to compare and rank the relevant efficacy indicators of acupuncture and massage in the treatment of KOA in order to provide clinical guidance for acupuncture and massage in the treatment of KOA.

At present, the efficacy indicators related to knee osteoarthritis are mainly clinical effective rate, VAS score, total Lysholm score, total WOMAC score and WOMAC stiffness, and joint function score. Therefore, in this study, combined with the previous relevant research experience, the effective rate and the above scores were used as efficacy evaluation indicators, and network meta-analysis was used to compare the efficacy of various acupuncture combined with massage alone, the above acupuncture therapy alone or this therapy combined with massage, and rank the different outcome indicators. This comparison not only involves the comparison with monotherapy but also allows the direct comparison of both groups as a combined method, both direct and indirect, making the results more evidence-based. This study involved 16 interventions, and the results showed that the top five regimens in the ranking of effective rate probability were floating needle+massage>needle knife+massage>silver needle+massage>electrothermal needle+massage>common acupuncture+massage; in terms of reducing VAS, the top three regimens in the ranking were common acupuncture+massage>needle knife+massage>warm needle+massage; in terms of reducing total Lysholm score, the top three regimens in the ranking were silver needle+massage>electroacupuncture+massage>needle knife+massage; in terms of reducing total WOMAC score, the top three regimens in the ranking were silver needle+massage>electrothermal needle+massage>common acupuncture+massage; in terms of reducing WOMAC stiffness score, the top three regimens in the ranking were warm needle+massage>silver needle+massage; in terms of reducing WOMAC joint function score, the top three regimens in the ranking were silver needle+massage>warm needle+massage>common acupuncture+massage. In the comparison of the six outcome measures included in this study, it was found that each treatment combination had a large difference in the optimal ranking among the different outcome measures, so it had some difficulties in selecting the optimal acupuncture and massage regimen. Acupuncture combined with massage was superior to acupuncture alone in improving multiple group comparisons of outcome measures of clinical effective rate, VAS score, WOMAC stiffness, and joint function score. At the same time, this study also found that the first three regimens with the best improvement of each outcome measure were a combination of acupuncture and massage. This may be an important enlightenment for the application of acupuncture and massage in the treatment of KOA, suggesting that clinicians can improve the clinical therapeutic effect of KOA through the combined use of acupuncture and massage. The effective rate, as the main outcome measure, is a comprehensive evaluation of the efficacy. Network meta-analysis shows that floating needle+massage may be the most effective intervention to improve the overall symptoms of KOA patients. Floating needle therapy can effectively improve the symptoms of KOA patients by sweeping away the subcutaneous tissue and changing the ion channel of cells. With massage, it can change the lower limb strength line and enhance the stability of joints [85, 86]. The most obvious symptoms in patients with KOA are pain and limited mobility. As an indicator of pain, VAS score can effectively reflect the degree of pain in patients with KOA. Common acupuncture+massage and needle knife+massage were better in improving VAS, which may indicate that the above two methods can be preferred when pain is predominant in KOA patients. Common acupuncture can reduce the levels of IL-1, IL-17, and other inflammatory factors to relieve pain and promote muscle, soft tissue, and other recovery. Needle knife therapy can release tendons and knots and regulate muscles to restore muscle and bone balance. On the basis of common acupuncture or needle knife, the combination with massage can enhance its analgesic effect [87, 88]. WOMAC-related scores and total Lysholm score directly involve or synthesize the performance of daily activities of KOA patients. The study found that the therapy with warming effect had better effect in improving the limitation of activities of patients. Silver needle+massage may be the best therapy for improving the limitation of activities of KOA patients. Silver needle therapy can promote the release of hormones such as T-AOC and SOD and, in combination with massage manipulation, can improve knee blood circulation to restore joint function [89, 90]. Therefore, the application of the above interventions should be customized according to the patient's characteristics and condition, and the probability ranking results are only for clinicians' reference. In this study, after removing the nonconventional massage, the results of network meta-analysis of each index in the literature were stable, which indicated that there may be no significant difference in the efficacy of different massage techniques in the treatment of knee osteoarthritis. However, due to the small number of literatures, after removing the literatures in terms of effective rate, there is less than one kind of intervention measure. Some outcome measures, such as WOMAC-related score, cannot be subjected to network meta-analysis after removing the literatures, and there may be some bias phenomenon. After the high-risk literatures were excluded in this study, the overall results were still robust, with some changes in VAS score, which had little impact on the overall results, indicating that the quality of the literatures included in this study was acceptable.

This study also has some limitations: The quality of the included studies needs to be improved; among 49 RCTs, only 24 reported the method of random allocation, and 2 mentioned the use of blind method, so there may be implementation bias. Because the inclusion and exclusion criteria are relatively strict, the RCTs of press needle, blade needle, movable needle, blood-letting puncture, and other methods are not included, so the above therapies are not statistically analyzed. The included studies are mostly short-term, small sample size RCTs and lack a perfect follow-up process; The clinical effective rate as a simple measurement, is most frequently used in clinical practice. However, the definition between studies may be greatly varied. It to some extent contributed to the heterogeneity of the results and therefore decreased the power of the evidence provided by the present study. The included literatures are in Chinese, the previous trial protocols are not disclosed in advance, and there is a possibility of selective reporting.

In summary, this network meta-analysis showed that the combination of acupuncture and massage has obvious advantages in the treatment of KOA, which can be used as one of the clinical program options. It has a better intervention effect of floating needle+massage in improving the overall symptoms of KOA patients, a better effect of common acupuncture+massage, needle knife+massage in improving the pain of KOA patients, and a better effect of silver needle+massage in improving the daily activities of KOA patients. In clinical practice, the appropriate treatment should be selected according to the patient's condition. Due to the limitation of the number and quality of included original studies, more double-blind, multicenter, large-sample, high-quality clinical trials are still needed for supplementary verification in order to provide stronger evidence support for acupuncture and massage in the treatment of KOA.

## Figures and Tables

**Table 1:** Basic characteristics of included studies.

Included studies	Mean age (years)		Sample size (M/F)		Mean disease duration (year)		Interventions		Duration/day	Follow-up or not	Outcome measures
	T	C	T	C	T	C	T	C			
Ye et al. [26]	61.03 ± 7.21	61.41 ± 7.12	23/23	24/22	4.41 ± 1.31	4.47 ± 1.24	Electrothermal needle+loosening	Loosening	4 weeks	No	①④
Ye et al. [27]	61 ± 7	62 ± 5	18/29	20/25	5.28 ± 2.75	5.65 ± 3.05	Heating needle+Maitland	Maitland	4 weeks	No	①④
Zhou [28]	40~70	40~70	20/30	20/30	14 d~7 years	19 d~6 years	Float needle+tuina	Tuina	14 d	No	①
Chao et al. [29]	61.38 ± 8.59	59.48 ± 9.26	3/18	5/16	21.3 ± 8.47 months	20.7 ± 9.35 months	Fire needle+Meridian-sinew manipulation	Meridian-sinew manipulation	2 weeks	Yes	②④
Lan [30]	53.91 ± 5.09	53.61 ± 5.38	33/27	35/25	—	—	Silver needle+tuina	Tuina	2 weeks	No	①③
Duning [31]	57.98 ± 9.27	58.94 ± 7.54	7/43	6/44	1.64 ± 1.41	1.60 ± 1.35	Beryllium needle+tuina	Tuina	20 d	No	①
Jiang et al. [32]	62.3 ± 5.8	60.5 ± 3.3	13/37	9/41	74.62 ± 5.68 months	75.10 ± 3.60 months	Electroacupuncture+tuina	Tuina	30 d	No	①
Hongyan et al. [33]	55.6 ± 2.9	54.9 ± 3.1	25/35	20/30	4.8 ± 1.6	4.4 ± 1.5	Warm needling+tuina	Tuina	20 d	No	①②
Yuanhong et al. [34]	52.68 ± 8.25	52.71 ± 9.35	11/19	13/17	1.65 ± 0.16	1.64 ± 0.83	Warm needle+Maitland	Maitland	4 weeks	No	②③
Wang [35]	57.91 ± 11.94	58.49 ± 11.20	23/55	19/59	4.07 ± 1.13	4.12 ± 1.07	Warm needling+tuina	Tuina	20 d	No	①②③
Huangke [36]	35~85	35~85	36	36	—	—	Warm needling+tuina	Tuina	20 d	No	①②③
Huang et al. [37]	55.72 ± 9.69	56.13 ± 9.84	39/81	78/22	4.72 ± 1.01	4.85 ± 1.18	Warm needling+tuina	Tuina	20 d	No	①②③
Qian et al. [38]	55.79 ± 7.86	59.84 ± 7.77	14/18	13/19	18.6 ± 5.3 months	19.7 ± 6 5 months	Warming needle+loosening	Loosening	20 d	No	①⑤
Chen [39]	54.82 ± 6.49	55.18 ± 6.20	23/17	25/15	4.35 ± 2.12	4.28 ± 2.09	Warm needling+tuina	Tuina	30 d	Yes	①②
Guan [40]	60.45 ± 5.86	59.65 ± 5.79	25/30	27/28	5.55 ± 2.26	5.65 ± 2.45	Warm needling+tuina	Tuina	4 weeks	No	①
Qiao [41]	62.39 ± 5.41	61.73 ± 5.06	19/31	17/25	4.79 ± 1.12	4.82 ± 1.03	Warm needling+tuina	Tuina	4 weeks	No	①⑤
Zhang et al. [42]	47.5 ± 7.1	46.3 ± 8.7	13/18	12/19	——————	——————	Needle knife + Tuina	Tuina	14d	No	①②③
Guibo and Mao [43]	63.96 ± 3.12	64.25 ± 2.92	20/20	22/18	5.95 ± 0.49	6.12 ± 0.54	Needle knife+tuina	Tuina	2 weeks	No	①
Wang and Lang [44]	58 ± 8	57 ± 10	16/21	19/19	7.01 ± 4.81	6.84 ± 4.21	Needle knife+Maitland	Maitland	4 weeks	Yes	①②③
Wang and Lang [45]	57.76 ± 5.23	58.28 ± 4.54	33/40	32/35	21.37 ± 10.42 months	22.21 ± 12.31 months	Needle knife+tuina	Tuina	3 weeks	No	①②
Ma [46]	64.5 ± 7.2	65.3 ± 8.6	12/23	13/22	5.7 ± 2.3	6.1 ± 2.7	Needle knife+tuina	Tuina	14 d	No	②③
Liangi [47]	43~64	47~63	13/17	12/18	3 months~15 years	2 months~10 years	Needle knife+tuina	Tuina	10 d	No	①
Liao [48]	59.02 ± 9.57	60.31 ± 9.86	14/26	17/23	8 months~18 years	8 months~18 years	Common acupuncture+tuina	Tuina	28 d	No	①②
Chao [49]	55.2 ± 8.5	55.8 ± 8.6	15/25	14/22	3.5 ± 1.3	3.6 ± 1.4	Common acupuncture+tuina	Tuina	20 d	No	①
Gao [50]	41.0 ± 9.4	40.0 ± 9.3	18/22	19/21	—	—	Common acupuncture+tuina	Tuina	30 d	No	⑤
Dong et al. [51]	56.28 ± 4.50	55.87 ± 5.66	15/25	17/23	14.23 ± 5.11	13.71 ± 4.58	Common acupuncture+Dong's massage	Dong's massage	20 d	No	①②④
Wang and Ma [52]	51.6 ± 3.49	52.1 ± 4.50	12/23	16/19	2.0 ± 1.8	2.3 ± 1.9	Catgut embedding needle knife+tuina	Common acupuncture	3 weeks	No	①②
Lin et al. [53]	59.8 ± 11.6	60.7 ± 12.1	19/31	22/28	6.9 ± 5.1	7.0 ± 5.3	Warm needling + Tuina	Common acupuncture	14 d	No	①
Li [54]	50.4 ± 7.8	51.2 ± 8.1	17/15	14/18	16.3 ± 5.9 months	17.1 ± 6.1 months	Warm needling+tuina	Common acupuncture	30 d	No	①②④
Xiong et al. [55]	65.23 ± 11.58	64.72 ± 12.47	19/27	21/24	5.76 ± 2.16	5.49 ± 2.37	Common acupuncture+tuina	Common acupuncture	20 d	No	①②④⑤
Huang et al. [56]	60.5 ± 7.4	58.7 ± 7.1	12/18	13/17	7.3 ± 2.1	6.9 ± 2.2	Common acupuncture+tuina	Common acupuncture	20 d	No	①
Ding [57]	67.58 ± 6.25	65.33 ± 7.46	9/17	12/18	7.08 ± 4.20	7.66 ± 4.80	Common acupuncture+tuina	Common acupuncture	8 weeks	No	②③
Chen et al. [58]	61.2 ± 9.3	60.1 ± 9.3	14/17	15/16	5.9 ± 2.7	5.4 ± 3.1	Electroacupuncture+Wei's maneuver	Electroacupuncture	6 weeks	No	①
Ma and Wu [59]	56.6 ± 11.9	57.2 ± 11.6	17/25	16/26	—	—	Electroacupuncture+tuina	Electroacupuncture	3 weeks	No	①②③
Zeng [60]	53.26 ± 6.71	53.30 ± 6.88	19/11	18/12	3.35 ± 1.01	3.37 ± 1.02	Needle knife+tuina	Needle knife	4 weeks	No	②
Li and Zhang [61]	58.52 ± 6.28	59.11 ± 5.46	22/28	21/29	4.84 ± 1.23	5.14 ± 2.03	Needle knife+tuina	Needle knife	3 weeks	Yes	①②
Zhu [62]	61.2 ± 5.8	60.8 ± 5.9	23/28	24/27	3.0 ± 0.5	3.2 ± 0.6	Needle knife+tuina	Needle knife	4 weeks	No	①
Li [63]	65.3 ± 5.7	67.0 ± 6.1	19/46	20/45	10.2 ± 2.5	9.8 ± 2.1	Warm needling+tuina	Warm needling	14 d	No	①②③
Shen et al. [64]	56.41 ± 5.27	57.12 ± 5.19	39/71	42/68	6.27 ± 2.05	6.33 ± 2.22	Warm needling+tuina	Warm needling	12 d	No	①②
Zhou et al. [65]	56.02 ± 3.37	56.01 ± 2.97	14/16	13/17	2.22 ± 0.45	2.34 ± 0.54	Warm needling+tuina	Warm needling	14 d	No	①②
Zhou et al. [66]	59.96 ± 6.33	59.66 ± 7.45	7/21	8/21	65.43 ± 30.75 months	66.34 ± 36.49 months	Warm needling+tuina	Warm needling	4 weeks	No	①②④⑤
Xiong and Chen [67]	59.5 ± 3.3	58.9 ± 3.8	37/43	40/38	3.5 ± 1.9	3.4 ± 2.1	Needle knife+tuina	Electroacupuncture	3 weeks	No	①②③
Zheng and Dong [68]	51.36 ± 1.45	51.84 ± 1.66	18/14	17/15	6.32 ± 1.59	6.48 ± 1.63	Fire needling+tuina	Warm needling+tuina	14 d	No	①②
Wang et al. [69]	65.3 ± 15.6	59.3 ± 14.7	10/14	8/16	3.4 ± 2.9	3.2 ± 2.8	Warm needling+tuina	Common acupuncture+tuina	30 d	No	②③
Lu et al. [70]	58.62 ± 13.46	57.21 ± 12.77	37/46	38/44	14.49 ± 8.63 months	15.35 ± 8.03 months	Needle knife+tuina	Common acupuncture+tuina	3 weeks	No	①③
Wang and Liu [71]	58.27 ± 3.85	67.31 ± 4.21	21/24	18/27	14.76 ± 5.75 months	16.21 ± 4.57 months	Needle knife+tuina	Common acupuncture+tuina	3 weeks	No	②
Chen et al. [72]	65.11 ± 5.22	65.11 ± 5.22	18/22	18/22	15.36 ± 6.25 months	15.44 ± 6.34 months	Needle knife+tuina	Common acupuncture+tuina	3 weeks	Yes	①
Zhang et al. [73]	53.91 ± 5.59	55.32 ± 7.29	18/24	20/24	4.58 ± 2.76	4.51 ± 2.86	Silver needling+tuina	Silver needle	3 weeks	Yes	④⑤
		56.11 ± 8.12		19/30		4.53 ± 2.82		Tuina			
Zhang et al. [74]	61.5 ± 7.64	63.4 ± 4.98	22/8	23/7	9.5 ± 1.2	8.8 ± 1.4	Common acupuncture+tuina	Common acupuncture	4 weeks	No	①②
		59.8 ± 7.25		18/12		9.2 ± 2.5		Tuina			

T: test group; C: control group; —: it was not mentioned. ^①^Total effective rate. ^②^VAS. ^③^Lysholm. ^④^Total WOMAC. ^⑤^WOMAC stiffness and joint function.

**(a):** 

Interventions	Float needle+massage	Needle knife+massage	Silver needle+massage	Electrothermal needle+massage	Common acupuncture+massage	Beryllium needle+massage	Warm needling+massage
Float needle+massage	0						
Needle knife+massage	2.62 (0.51, 13.54)	0					
Silver needle+massage	2.46 (0.28, 21.66)	0.94 (0.18, 4.98)	0				
Electrothermal needle+massage	4.84 (0.79, 29.81)	1.85 (0.58, 5.92)	1.97 (0.31, 12.45)	0			
Common acupuncture+massage	5.45 (1.05, 28.27)^1)^	2.08 (1.01, 4.27)^1)^	2.22 (0.42, 11.83)	1.12 (0.35, 3.63)	0		
Beryllium needle+massage	6.27 (0.78, 50.15)	2.39 (0.51, 11.17)	2.55 (0.31, 20.87)	1.30 (0.23, 7.29)	1.15 (0.25, 5.41)	0	
Warm needling+massage	7.60 (1.59, 36.34)^1)^	2.90 (1.43, 5.86)^1)^	3.09 (0.63, 15.23)	1.57 (0.55, 4.50)	1.39 (0.69, 2.82)	1.21 (0.28, 5.21)	0
Electroacupuncture+massage	8.05 (0.99, 65.28)	3.07 (0.70, 13.44)	3.28 (0.40, 27.16)	1.66 (0.29, 9.51)	1.48 (0.32, 6.88)	1.28 (0.17, 9.62)	1.06 (0.24, 4.65)
Fire needle+massage	9.85 (1.19, 81.35)^1)^	3.75 (0.77, 18.27)	4.01 (0.47, 33.84)	2.03 (0.35, 11.89)	1.81 (0.37, 8.80)	1.57 (0.21, 12.00)	1.30 (0.31, 5.35)
Catgut embedding needle knife+massage	10.30 (1.08, 97.90)^1)^	3.93 (0.69, 22.39)	4.19 (0.43, 40.67)	2.13 (0.31, 14.68)	1.89 (0.35, 10.21)	1.64 (0.19, 14.52)	1.36 (0.26, 7.19)
Needle knife	14.36 (1.95, 105.68)^1)^	5.47 (1.76, 17.04)^1)^	5.84 (0.78, 44.01)	2.96 (0.58, 15.08)	2.64 (0.69, 10.11)	2.29 (0.34, 15.52)	1.89 (0.50, 7.19)
Massage	16.00 (3.49, 73.41)^1)^	6.10 (3.31, 11.24)^1)^	6.51 (1.38, 30.79)^1)^	3.30 (1.23, 8.90)^1)^	2.94 (1.57, 5.49)^1)^	2.55 (0.62, 10.49)	2.11 (1.47, 3.02)^1)^
Warm needle	17.85 (3.33, 95.74)^1)^	6.81 (2.68, 17.27)^1)^	7.26 (1.32, 40.05)^1)^	3.69 (1.09, 12.46)^1)^	3.28 (1.29, 8.32)^1)^	2.85 (0.59, 13.83)	2.35 (1.28, 4.32)^1)^
Common acupuncture	27.46 (4.88, 154.56)^1)^	10.47 (3.96, 27.68)^1)^	11.17 (1.93, 64.60)^1)^	5.67 (1.57, 20.47)^1)^	5.04 (2.11, 12.04)^1)^	4.38 (0.86, 22.40)	3.61 (1.57, 8.33)^1)^
Electroacupuncture	35.16 (4.05, 305.17)^1)^	13.41 (2.91, 61.67)^1)^	14.31 (1.61, 126.88)^1)^	7.26 (1.17, 45.04)^1)^	6.46 (1.28, 32.47)^1)^	5.61 (0.70, 45.11)	4.63 (0.96, 22.31)

**(b):** 

Interventions	Electroacupuncture+massage	Fire needle+massage	Catgut embedding needle knife+massage	Needle knife	Massage	Warm needle	Common acupuncture	Electroacupuncture
Float needle+massage								
Needle knife+massage								
Silver needle+massage								
Electrothermal needle+massage								
Common acupuncture+massage								
Beryllium needle+massage								
Warm needling+massage								
Electroacupuncture+massage	0							
Fire needle+massage	1.22 (0.16, 9.48)	0						
Catgut embedding needle knife+massage	1.28 (0.14, 11.38)	1.05 (0.12, 9.33)	0					
Needle knife	1.78 (0.28, 11.48)	1.46 (0.21, 10.22)	1.39 (0.17, 11.15)	0				
Massage	1.99 (0.47, 8.34)	1.62 (0.38, 7.01)	1.55 (0.30, 8.16)	1.11 (0.31, 4.05)	0			
Warm needle	2.22 (0.45, 10.96)	1.81 (0.39, 8.48)	1.73 (0.29, 10.24)	1.24 (0.29, 5.40)	1.12 (0.55, 2.26)	0		
Common acupuncture	3.41 (0.66, 17.58)	2.79 (0.54, 14.44)	2.67 (0.63, 11.31)	1.91 (0.43, 8.53)	1.72 (0.76, 3.88)	1.54 (0.55, 4.32)	0	
Electroacupuncture	4.37 (1.34, 14.18)^1)^	3.57 (0.43, 29.66)	3.42 (0.36, 32.32)	2.45 (0.37, 16.41)	2.20 (0.47, 10.17)	1.97 (0.36, 10.64)	1.28 (0.23, 7.16)	0

The difference between the two groups had statistical significance (^1)^*P* < 0.05).

**Table 3:** Network meta-analysis of VAS.

Interventions	Common acupuncture+massage	Needle knife+massage	Warm needling+massage	Electroacupuncture+massage	Fire needle+massage	Catgut embedding needle knife+massage
Common acupuncture+massage	0					
Needle knife+massage	-0.08 (-1.47, 1.32)	0				
Warm needling+massage	-0.17 (-1.20, 0.85)	-0.09 (-1.33, 1.14)	0			
Electroacupuncture+massage	0.00 (-3.14, 3.14)	0.08 (-2.73, 2.89)	0.17 (-2.89, 3.24)	0		
Fire needle+massage	-0.58 (-2.29, 1.13)	-0.50 (-2.30, 1.29)	-0.41 (-1.89, 1.08)	-0.58 (-3.91, 2.75)	0	
Catgut embedding needle knife+massage	-1.14 (-3.46, 1.18)	-1.06 (-3.64, 1.52)	-0.97 (-3.35, 1.41)	-1.14 (-4.95, 2.67)	-0.56 (-3.32, 2.20)	0
Electroacupuncture	-1.60 (-4.04, 0.84)	-1.52 (-3.52, 0.48)	-1.43 (-3.77, 0.92)	-1.60 (-3.57, 0.37)	-1.02 (-3.71, 1.67)	-0.46 (-3.72, 2.81)
Massage	-1.67 (-2.62, -0.72)^1)^	-1.59 (-2.61, -0.57)^1)^	-1.50 (-2.19, -0.81)^1)^	-1.67 (-4.66, 1.32)	-1.09 (-2.57, 0.39)	-0.53 (-2.90, 1.84)
Warm needle	-1.84 (-3.25, -0.42)^1)^	-1.76 (-3.31, -0.20)^1)^	-1.66 (-2.65, -0.68)^1)^	-1.84 (-5.05, 1.38)	-1.25 (-3.03, 0.52)	-0.69 (-3.26, 1.88)
Needle knife	-1.94 (-3.91, 0.02)	-1.86 (-3.25, -0.48)^1)^	-1.77 (-3.62, 0.08)	-1.94 (-5.07, 1.19)	-1.36 (-3.63, 0.91)	-0.80 (-3.73, 2.13)
Common acupuncture	-1.88 (-2.93, -0.84)^1)^	-1.80 (-3.35, -0.26)^1)^	-1.71 (-2.89, -0.53)^1)^	-1.88 (-5.09, 1.32)	-1.30 (-3.12, 0.52)	-0.74 (-2.81, 1.33)
Interventions	Electroacupuncture	Massage	Warm needle	Needle knife	Common acupuncture	
Common acupuncture+massage						
Needle knife+massage						
Warm needling+massage						
Electroacupuncture+massage						
Fire needle+massage						
Catgut embedding needle knife+massage						
Electroacupuncture	0					
Massage	-0.07 (-2.32, 2.17)	0				
Warm needle	-0.24 (-2.77, 2.30)	-0.16 (-1.34, 1.01)	0			
Needle knife	-0.34 (-2.77, 2.09)	-0.27 (-1.99, 1.45)	-0.11 (-2.19, 1.98)	0		
Common acupuncture	-0.28 (-2.81, 2.24)	-0.21 (-1.37, 0.95)	-0.05 (-1.57, 1.48)	0.06 (-2.01, 2.13)	0	

The difference between the two groups had statistical significance (^1)^*P* < 0.05).

**(a):** 

Interventions	Silver needle+massage	Electroacupuncture+massage	Needle knife+massage	Warm needling+massage	Massage
Silver needle+massage	0				
Electroacupuncture+massage	2.63 (-3.23, 8.50)	0			
Needle knife+massage	7.09 (1.48, 12.71)^1)^	4.46 (2.77, 6.15)^1)^	0		
Warm needling+massage	13.10 (7.74, 18.45)^1)^	10.46 (7.89, 13.03)^1)^	6.00 (4.07, 7.94)^1)^	0	
Massage	18.81 (13.51, 24.11)^1)^	16.18 (13.67, 18.69)^1)^	11.72 (9.86, 13.58)^1)^	5.71 (4.96, 6.47)^1)^	0
Electroacupuncture	19.63 (13.95, 25.32)^1)^	17.00 (15.57, 18.43)^1)^	12.54 (11.65, 13.43)^1)^	6.54 (4.41, 8.67)^1)^	0.82 (-1.24, 2.88)
Warm needle	21.63 (15.45, 27.80)^1)^	18.99 (14.98, 23.01)^1)^	14.53 (10.89, 18.18)^1)^	8.53 (5.44, 11.62)^1)^	2.82 (-0.36, 5.99)
Common acupuncture+massage	25.90 (20.24, 31.55)^1)^	23.26 (20.50, 26.03)^1)^	18.80 (16.62, 20.99)^1)^	12.80 (10.89, 14.71)^1)^	7.09 (5.11, 9.06)^1)^
Common acupuncture	44.11 (37.04, 51.17)^1)^	41.47 (36.42, 46.52)^1)^	37.01 (32.25, 41.77)^1)^	31.01 (26.37, 35.65)^1)^	25.30 (20.63, 29.96)^1)^

**(b):** 

Interventions	Electroacupuncture	Warm needle	Common acupuncture+massage	Common acupuncture
Silver needle+massage				
Electroacupuncture+massage				
Needle knife+massage				
Warm needling+massage				
Massage				
Electroacupuncture	0			
Warm needle	1.99 (-1.76, 5.74)	0		
Common acupuncture+massage	6.26 (3.90, 8.62)^1)^	4.27 (0.64, 7.90)^1)^	0	
Common acupuncture	24.47 (19.63, 29.32)^1)^	22.48 (16.91, 28.05)^1)^	18.21 (13.98, 22.44)^1)^	0

The difference between the two groups had statistical significance (^1)^*P* < 0.05).

## Data Availability

All data relevant to the study are included in the article or uploaded as online supplemental information. The data used in this review was collected from the forty-nine eligible studies and therefore available in the public domain.

## Supplementary Materials

Supplementary Materials Supplementary Materials Search strategies for RCTs (Table S1; pages 2-3); risk of bias summary (Figure S1; page 4): the results of total WOMAC score, WOMAC stiffness, and joint function scores (Figure S2-9; Table S2-4; pages 5-11). The results of sensitivity analyses (Figure S10-21; Table S3-10; pages 12-28). The results of subgroup analyses (Figure S22-27; Table S11-16; pages 29-37). Introduction of the interventions involved in this study (Table S17; pages 38-39). The checklist of the PRISMA for network meta-analysis (Table S18; pages 40-44).
