# Supplementary material for: Systematic Review and Network Meta-analysis of Acupuncture Combined with Massage in Treating Knee Osteoarthritis
Source: Biomed Res Int. 2022 Aug 13;2022:4048550. doi: 10.1155/2022/4048550 (PMC9392627; doi:10.1155/2022/4048550)
Supplement: Supplementary Materials — Search strategies for RCTs (Table S1; pages 2-3); risk of bias summary (Figure S1; page 4): the results of total WOMAC score, WOMAC stiffness, and joint function scores (Figure S2-9; Table S2-4; pages 5-11). The results of sensitivity analyses (Figure S10-21; Table S3-10; pages 12-28). The results of subgroup analyses (Figure S22-27; Table S11-16; pages 29-37). Introduction of the interventions involved in this study (Table S17; pages 38-39). The checklist of the PRISMA for network meta-analysis (Table S18; pages 40-44). [file 4048550.f1.pdf]

Systematic Review and Network Meta-analysis of Acupuncture Combined with Massage in Treating Knee Osteoarthritis

Zhen Wang, Congan Wang, Yongquan Wang, Xujie Li, Ziyang Zhou, Lijuan Zhang, Miaoxiu Li, Yankun Pan, Tiyong Jiao , Xiaoyun Shi,Qing Liu.

Supplementary materials

**Table S1**	**Search Strategies of Network Meta-analysis**	**Page2-3**
**Figure S1**	**Risk of bias summary**	**page4**
**Figure S2-S9;<br>Table S2-S4**	**The results of total WOMAC,<br>WOMAC stiffness and joint function scores**	**Page5-11**
**Figure S10-S21;<br>Table S3-S10**	**The results of sensitivity analyses**	**Page12-28**
**Figure S22-S27;<br>Table S11-S16**	**The results of subgroup analysis**	**Page29-37**
**Table S17**	**Introduction to different acupuncture and massage therapies**	**Page38-39**
**Table S18**	**The checklist of the PRISMA for network meta-analysis**	**Page40-44**

Table S1 Search Strategies of Network Meta-analysis

Pubmed(Search date: July 15, 2021)

((((((Osteoarthritis, Knee[MeSH Terms]) OR (Knee Osteoarthritides[Title/Abstract])) OR (Knee Osteoarthritis[Title/Abstract])) OR (Osteoarthritis of Knee[Title/Abstract])) OR (Osteoarthritis of the Knee[Title/Abstract])) AND ((((((((((((((((((((((((((Acupuncture[MeSH Terms]) OR (Acupuncture[Title/Abstract])) OR (Pharmacopuncture[Title/Abstract])) OR (the warming acupuncture[Title/Abstract])) OR (warm acupuncture[Title/Abstract])) OR (warm needling acupuncture[Title/Abstract])) OR (Electroacupuncture[MeSH Terms])) OR (Electroacupuncture[Title/Abstract])) OR (fire needle[Title/Abstract])) OR (Acupuncture Therapy[MeSH Terms])) OR (Acupuncture Therapy[Title/Abstract])) OR (Acupuncture Treatment[Title/Abstract])) OR (Acupuncture Treatments[Title/Abstract])) OR (Pharmacoacupuncture Treatment[Title/Abstract])) OR (Pharmacoacupuncture Therapy[Title/Abstract])) OR (Acupotomy[Title/Abstract])) OR (Acupotomies[Title/Abstract])) OR (Water acupotomy therapy[Title/Abstract])) OR (Silver needle[Title/Abstract])) OR (Bloodletting acupuncture[Title/Abstract])) OR (Puncture cupping[Title/Abstract])) OR (Floating needle[Title/Abstract])) OR (Needles[MeSH Terms])) OR (Needles[Title/Abstract])) OR (Hypodermic Needles[Title/Abstract])) OR (Hypodermic Needle[Title/Abstract]))) AND (((((((((Tuina[Title/Abstract]) OR (Osteopathy[MeSH Terms])) OR (Osteopathy[Title/Abstract])) OR (Zone Therapy[Title/Abstract])) OR (Zone Therapies[Title/Abstract])) OR (Massage Therapy[Title/Abstract])) OR (Massage Therapies[Title/Abstract])) OR (massotherapy[Title/Abstract])) OR (Joint mobilization[Title/Abstract]))

Cochrane library(Search date: July 15, 2021)

#1 'Knee Osteoarthritis':ti,ab,kw OR 'Osteoarthritis of Knee':ti,ab,kw OR 'Osteoarthritis of the Knee':ti,ab,kw

#2 Acupuncture:ti,ab,kw OR Pharmacopuncture:ti,ab,kw OR the warming acupuncture:ti,ab,kw OR warm acupuncture:ti,ab,kw OR warm needling acupuncture:ti,ab,kw OR Electroacupuncture:ti,ab,kw OR fire needle:ti,ab,kw OR Acupotomy:ti,ab,kw OR Water acupotomy therapy:ti,ab,kw OR Silver needle:ti,ab,kw OR Bloodletting acupuncture:ti,ab,kw OR Puncture cupping:ti,ab,kw OR Floating needle:ti,ab,kw OR Needles:ti,ab,kw

#3 Tuina:ti,ab,kw OR Massage:ti,ab,kw OR Zone Therapy:ti,ab,kw OR massotherapy:ti,ab,kw OR Joint mobilization:ti,ab,kw

#1 AND #2 AND #3

Web of science(Search date: July 15, 2021)

#3 AND #2 AND #1

#3 TS=(Tuina OR Massage OR Zone Therapy OR massotherapy OR Joint mobilization)

#2 TS=(Acupuncture OR Pharmacopuncture OR the warming acupuncture OR warm acupuncture OR warm needling OR Electroacupuncture OR fire needle OR Acupotomy OR Water acupotomy therapy OR Silver needle OR Bloodletting acupuncture OR Floating needle OR Needle OR Needles)

#1 TS=(Knee Osteoarthritis OR Osteoarthritis of Knee OR Osteoarthritis of the Knee)

EMbase(Search date: July 15, 2021)

#3 AND #2 AND #1
#3 Tuina:ab,ti OR Massage:ab,ti OR Zone Therapy:ab,ti OR massotherapy:ab,ti OR Joint mobilization:ab,ti OR
#2 Acupuncture:ab,ti OR Pharmacopuncture:ab,ti OR the warming acupuncture:ab,ti OR warm acupuncture:ab,ti OR warm needling:ab,ti OR Electroacupuncture:ab,ti OR fire needle:ab,ti OR Acupotomy:ab,ti OR Water acupotomy therapy:ab,ti OR Silver needle:ab,ti OR Bloodletting acupuncture:ab,ti OR Floating needle:ab,ti OR Needle:ab,ti OR Needles:ab,ti
#1 'Knee Osteoarthritis':ab,ti OR 'Osteoarthritis of Knee':ab,ti OR 'Osteoarthritis of the Knee':ab,ti

China National Knowledge Infrastructure (CNKI) (Search date: July 15, 2021)

(SU=膝骨关节炎 OR SU=膝关节骨性关节炎 OR SU=膝关节骨关节病 OR SU=膝关节退行性关节炎 OR SU=膝痹 OR SU=膝痛症) AND (SU=针灸 OR SU=电针 OR SU=温针 OR SU=针刺 OR SU=动筋针 OR SU=火针 OR SU=针刀 OR SU=刃针 OR SU=刺络放血 OR SU=浮针 OR SU=针) AND (SU=推拿 OR SU=按摩 OR SU=整脊 OR SU=手法 OR SU=理筋)

VIP Database(Search date: July 15, 2021)

M=(膝骨关节炎 OR 膝关节骨性关节炎 OR 膝关节骨关节病 OR 膝关节退行性关节炎 OR 膝痹 OR 膝痛症) AND M=(针灸 OR 电针 OR 温针 OR 针刺 OR 动筋针 OR 火针 OR 针刀 OR 刃针 OR 刺络放血 OR 浮针 OR 针) AND M=(推拿 OR 按摩 OR 整脊 OR 手法 OR 理筋)

Chinese Biomedical Literature Database (CBM)(Search date: July 15, 2021)

(膝骨关节炎[标题] OR 膝关节骨性关节炎[标题] OR 膝关节骨关节病[标题] OR 膝关节退行性关节炎[标题] OR 膝痹[标题] OR 膝痛症[标题]) AND (针灸[摘要] OR 电针[摘要] OR 温针[摘要] OR 针刺[摘要] OR 动筋针[摘要] OR 火针[标题] OR 针刀[摘要] OR 刃针[摘要] OR 刺络放血[摘要] OR 浮针[摘要] OR 针[摘要]) AND (推拿[摘要] OR 按摩[摘要] OR 整脊[摘要] OR 手法[摘要] OR 理筋[摘要])

Wanfang databases(Search date: July 15, 2021)

主题:(膝骨关节炎 or 膝关节骨性关节炎 or 膝关节骨关节病 or 膝关节退行性关节炎 or 膝痹 or 膝痛症) and 主题:(针灸 or 电针 or 温针 or 针刺 or 动筋针 or 火针 or 针刀 or 刃针 or 刺络放血 or 浮针 or 针) and 主题:(推拿 or 按摩 or 整脊 or 手法 or 理筋)

Figure S1 Risk of bias summary

	Random sequence generation (selection bias)	Allocation concealment (selection bias)	Blinding of participants and personnel (performance bias)	Blinding of outcome assessment (detection bias)	Incomplete outcome data (attrition bias)	Selective reporting (reporting bias)	Other bias
Chen Chaowei 2018	+	?	?	?	+	+	?
Chen Haitao 2019	?	?	?	?	+	+	?
Chen Lanjun 2020	+	?	?	?	+	+	?
Chen Yanping 2019	+	?	?	?	+	+	?
Deng Ning 2012	+	?	?	?	+	+	?
Ding Yiqun 2010	?	?	?	?	+	+	?
Dong Linlin 2020	+	?	?	?	+	+	?
Gao bo 2018	?	?	?	?	+	+	?
Guan Xiangxin 2019	?	?	?	?	+	+	?
Huang Dongliang 2020	−	?	?	?	+	+	?
Huang Ke 2013	+	?	?	?	+	+	?
Huang Panyin 2007	?	?	?	?	+	+	?
Jiang Shengyun 2014	+	?	?	?	+	+	?
Liang Wei 2012	−	?	?	?	+	+	?
Liao Chunhong 2015	?	?	?	?	+	+	?
Li Chao 2015	−	?	?	?	+	+	?
Li Chao 2020	+	?	?	?	+	+	?
Li Jijie 2019	−	?	?	?	+	+	?
Lin Yuanfang 2012	+	?	−	?	+	+	?
Li Shuqiang 2017	?	?	?	?	+	+	?
Li Xia 2017	?	?	?	?	+	+	?
Lu Kaixu 2014	−	?	?	?	+	+	?
Ma Minghua 2020	+	?	?	?	+	+	?
Ma Youmeng 2016	?	?	?	?	+	+	?
Ning Yu 2013	+	?	?	?	+	+	?
Qiao Lei 2017	−	?	?	?	+	+	?
Qiu Xiuyu 2020	+	?	?	?	+	+	?
Shen Linxing 2020	+	?	?	?	+	+	?
Wang Feng 2017	−	?	?	?	+	+	?
Wang Haoran 2019	?	?	?	?	+	+	?
Wang Lijun 2021	?	?	?	?	+	+	?
Wang Luodan 2015	+	?	?	?	+	+	?
Wang Nan 2020	+	?	?	?	+	+	?
Xiong Peifang 2018	?	?	?	?	+	+	?
Xiong Xueqiong 2014	+	?	−	?	+	+	?
Xu Hongyan 2018	−	?	?	?	+	+	?
Xu Yuanhong 2016	?	?	?	?	+	+	?
Ye Bihong 2018	+	?	?	?	+	+	?
Ye Bihong 2019	+	?	?	?	+	+	?
Yu Guibo 2020	?	?	?	?	+	+	?
Zeng Yunyan 2018	+	?	?	?	+	+	?
Zhang Heng 2018	+	?	?	?	+	+	?
Zhang Lei 2019	+	?	?	?	+	+	?
Zhang Yongquan 2021	+	?	?	?	+	+	?
Zheng Desong 2017	+	?	?	?	+	+	?
Zhou Haitao 2011	?	?	?	?	+	+	?
Zhou Hao 2021	−	?	?	?	+	+	?
Zhou Ling 2017	−	?	?	?	+	+	?
Zhu Qinghui 2020	+	?	?	?	+	+	?

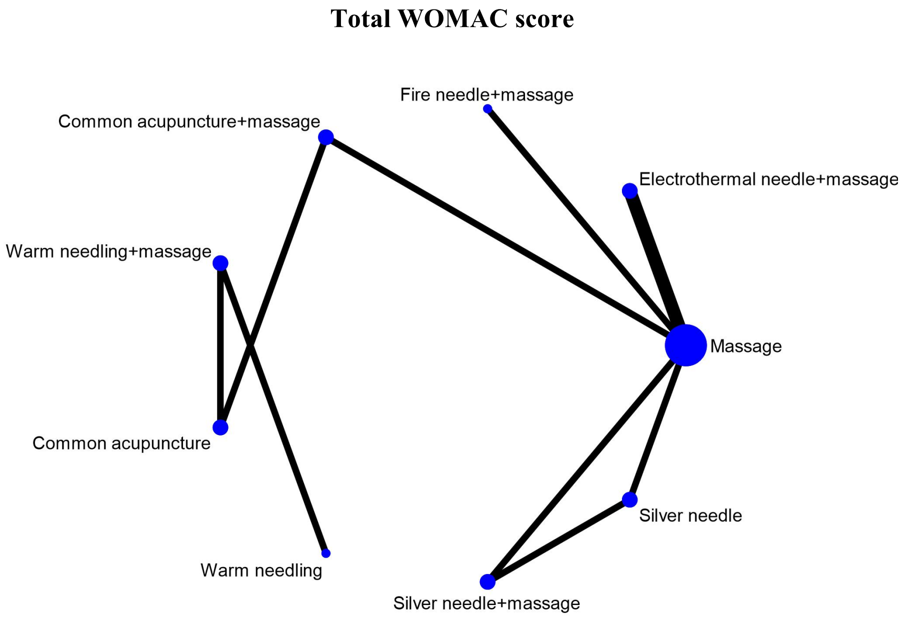

Figure S2 Network relationship diagram of total WOMAC score

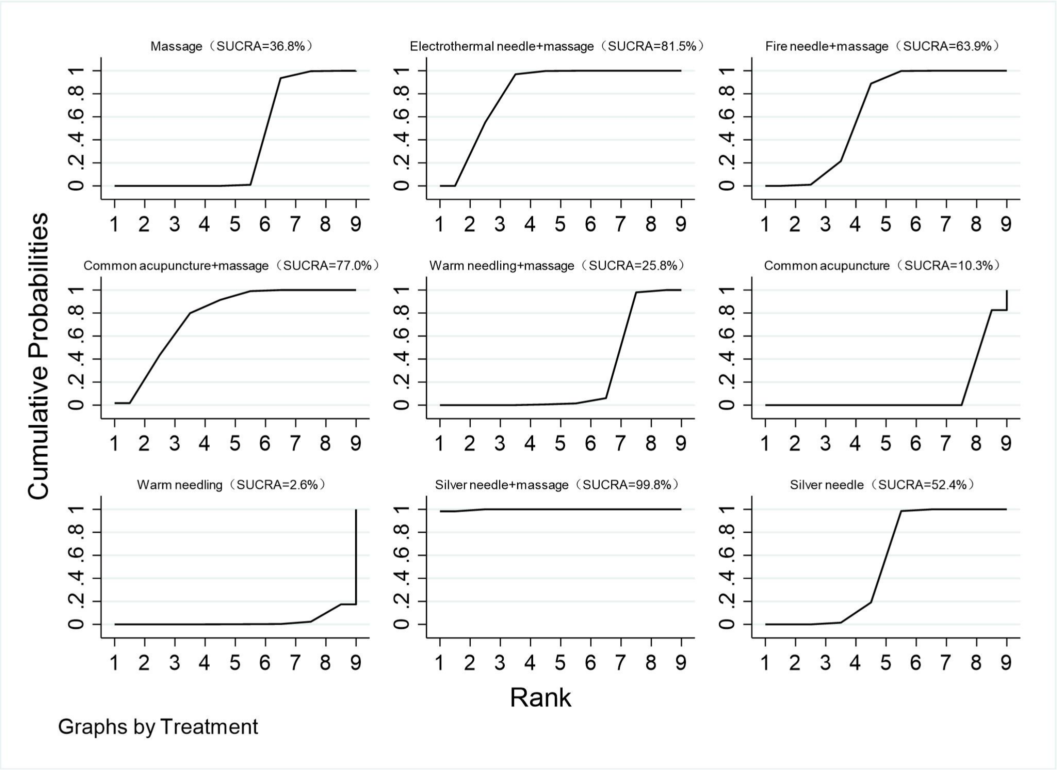

Figure S3 Sucra of total WOMAC score

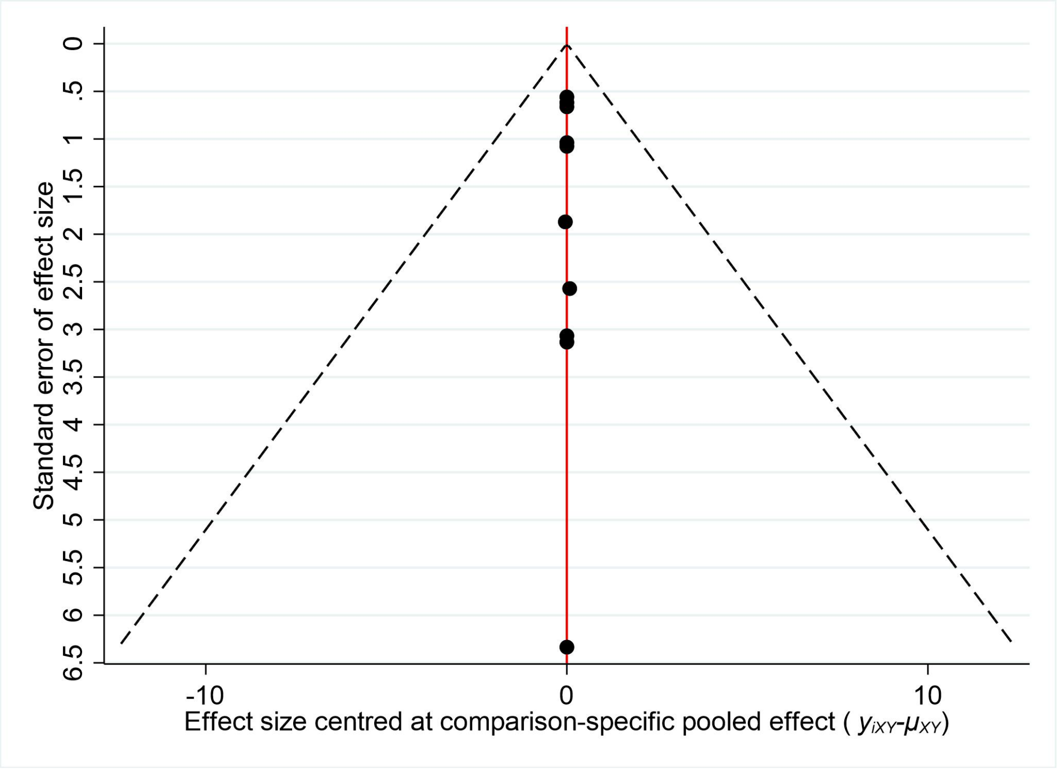

Figure S4 The funnel plot of total WOMAC score

Table S2 Network Meta-analysis of Total WOMAC Score

Interventions	Silver needle + massage	Electrothermal needle + massage	Common acupuncture + massage	Fire needle + massage	Silver needle
Silver + massage	0				
Electrothermal needle + massage	-6.21 (-9.37,-3.05)[1)]	0			
Common acupuncture + massage	-6.83 (-12.94,-0.73)[1)]	-0.62 (-7.32,6.08)	0		

Fire needle + massage	-9.58 (-11.89,-7.27)[1)]	-3.37 (-6.96,0.23)	-2.75 (-9.09,3.60)	0	
Silver needle	-11.00 (-12.21,-9.79)[1)]	-4.79 (-8.02,-1.55)[1)]	-4.17 (-10.31,1.98)	-1.42 (-3.83,0.99)	0
Massage	-14.10 (-15.20,-13.00)[1)]	-7.89 (-10.85,-4.92)[1)]	-7.27 (-13.27,-1.26)[1)]	-4.52 (-6.56,-2.48)[1)]	-3.10 (-4.40,-1.80)[1)]
Warm needling + massage	-20.94 (-29.85,-12.03)[1)]	-14.73 (-24.05,-5.40)[1)]	-14.10 (-20.59,-7.61)[1)]	-11.36 (-20.43,-2.28)[1)]	-9.94 (-18.88,-1.00)[1)]
Common acupuncture	-27.63 (-36.29,-18.97)[1)]	-21.42 (-30.50,-12.33)[1)]	-20.79 (-26.93,-14.66)[1)]	-18.05 (-26.87,-9.22)[1)]	-16.63 (-25.31,-7.94)[1)]
Warm needle	-33.79 (-49.07,-18.51)[1)]	-27.58 (-43.11,-12.05)[1)]	-26.95 (-40.96,-12.94)[1)]	-24.21 (-39.59,-8.83)[1)]	-22.79 (-38.09,-7.49)[1)]

Table 6 continued

Interventions	Massage	Warm needling + massage	Common acupuncture	Warm needle
Silver + massage				
Electrothermal needle + massage				
Common acupuncture + massage				
Fire needle + massage				
Silver needle				
Massage	0			
Warm needling + massage	-6.84 (-15.68,2.00)	0		
Common acupuncture	-13.53 (-22.12,-4.94)[1)]	-6.69 (-8.80,-4.58)[1)]	0	
Warm needle	-19.69 (-34.93,-4.44)[1)]	-12.85 (-25.27,-0.43)[1)]	-6.16 (-18.75,6.44)	0

Note: The difference between the two groups had statistical significance [1)] $P < 0.05$.

WOMAC stiffness and joint function scores

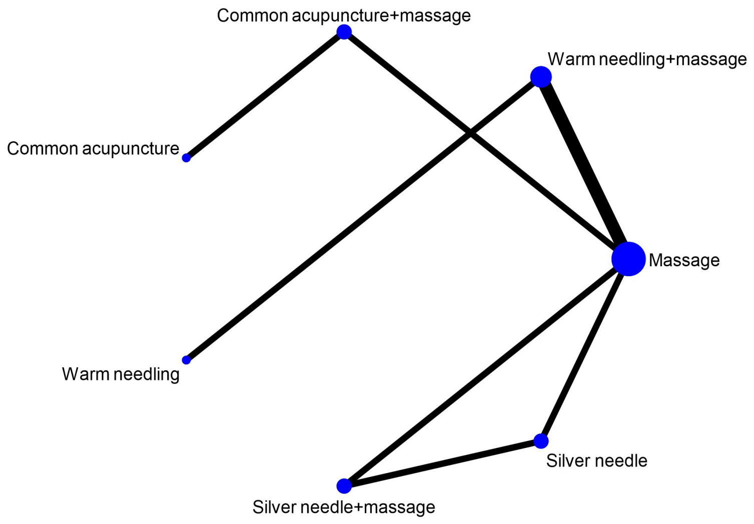

Figure S5 Network relationship diagram of WOMAC stiffness and joint function scores

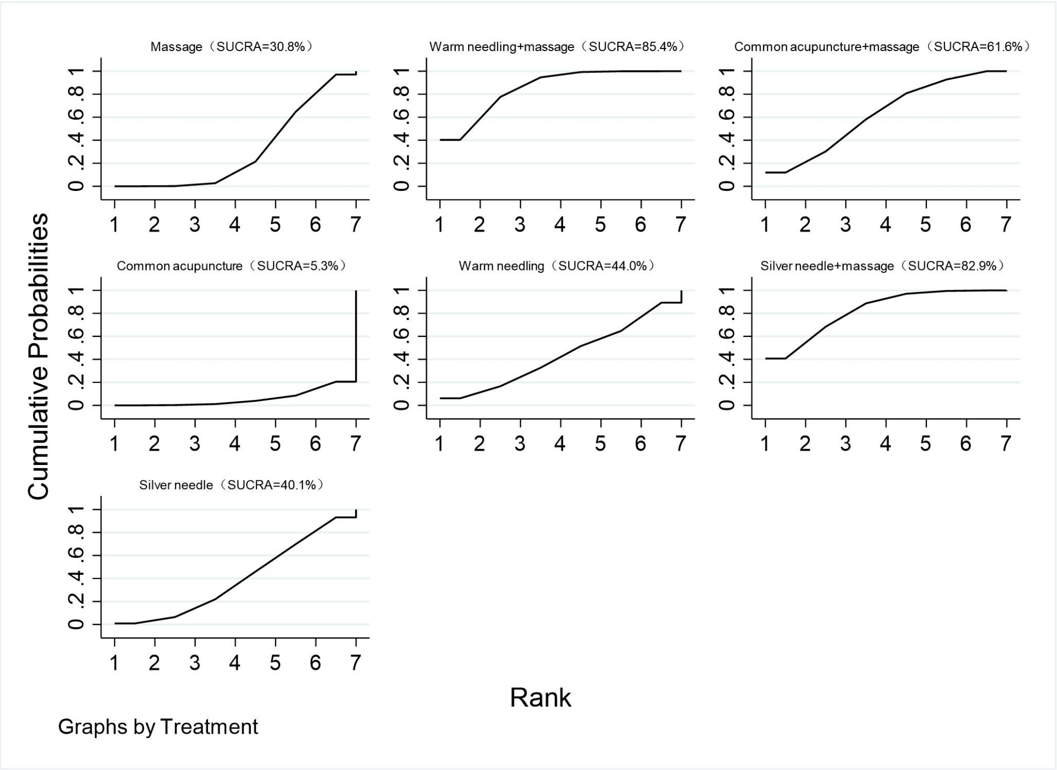

Figure S6 Sucra of WOMAC stiffness score

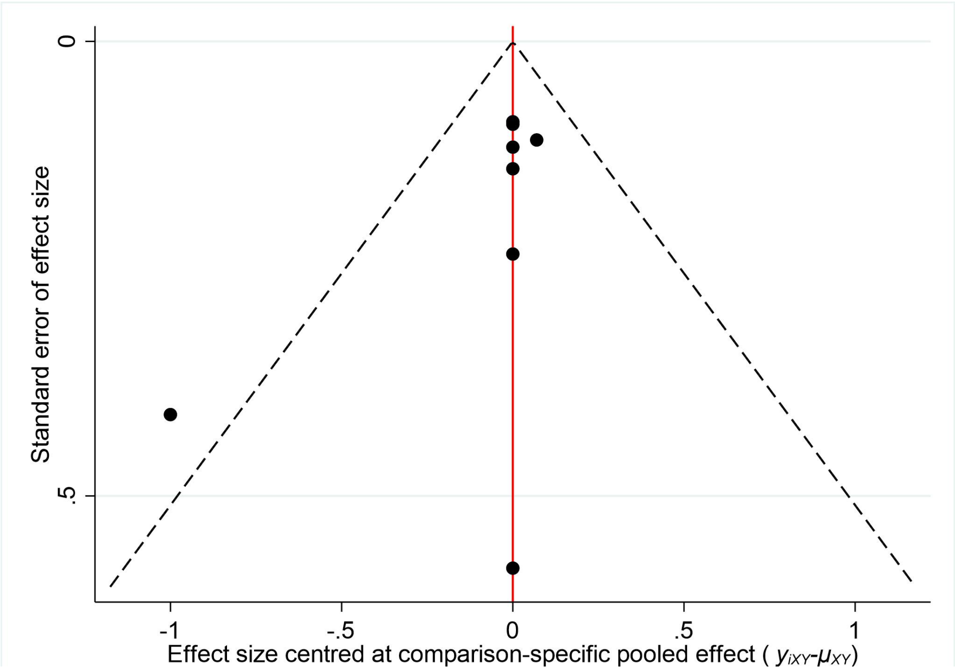

Figure S7 The funnel plot of WOMAC stiffness score

Table S3 Network Meta-analysis of WOMAC Stiffness Scores

Interventions	Warm needling + massage	Silver needle + massage	Common acupuncture + massage	Warm needle	Silver needle	Massage	Common acupuncture
Warm needling + massage	0						
Silver needle + massage	-0.06 (-1.81,1.68)	0					
Common acupuncture + massage	-0.66 (-2.39,1.07)	-0.60 (-2.55,1.35)	0				
Warm needle	-1.18 (-2.95,0.59)	-1.12 (-3.61,1.37)	-0.52 (-3.00,1.96)	0			
Silver needle	-1.26 (-3.00,0.48)	-1.20 (-2.57,0.17)	-0.60 (-2.54,1.34)	-0.08 (-2.56,2.40)	0		
Massage	-1.46 (-2.52,-0.40)[1)]	-1.40 (-2.79,-0.01)[1)]	-0.80 (-2.17,0.57)	-0.28 (-2.35,1.78)	-0.20 (-1.58,1.18)	0	
Common acupuncture	-2.84 (-5.09,-0.59)[1)]	-2.78 (-5.20,-0.36)[1)]	-2.18 (-3.62,-0.74)[1)]	-1.66 (-4.52,1.20)	-1.58 (-4.00,0.84)	-1.38 (-3.36,0.60)	0

Note: The difference between the two groups had statistical significance [1)] $P < 0.05$.

The Above is the WOMAC stiffness score

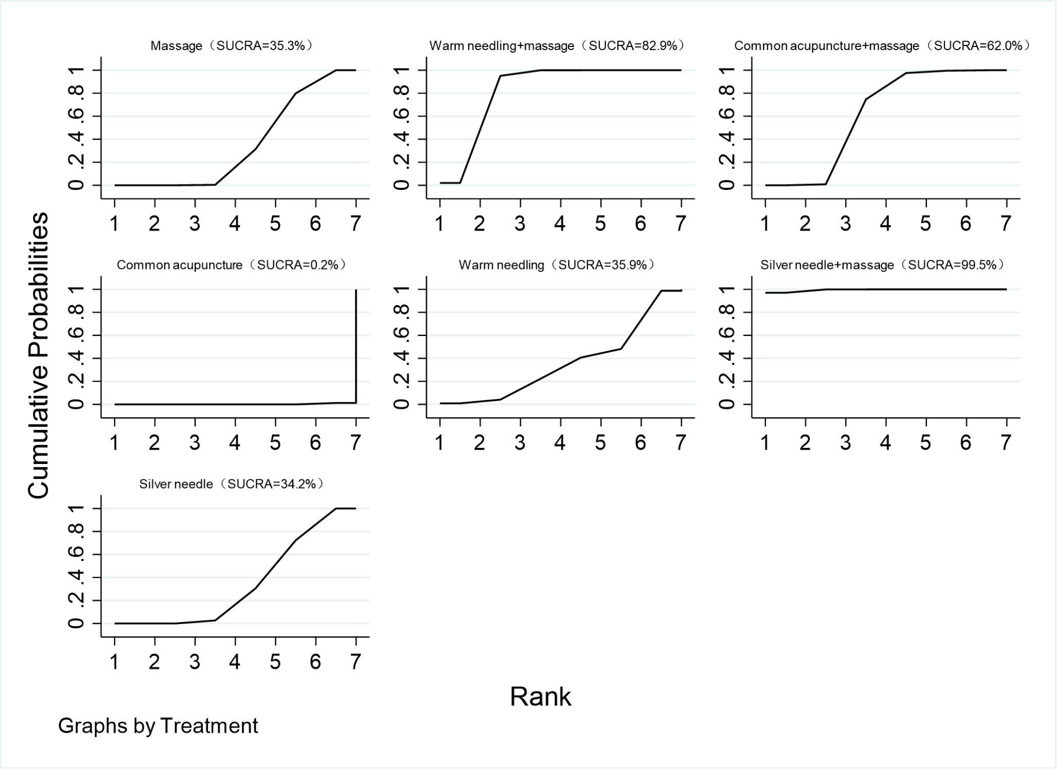

Figure S8 Sucra of WOMAC joint function score

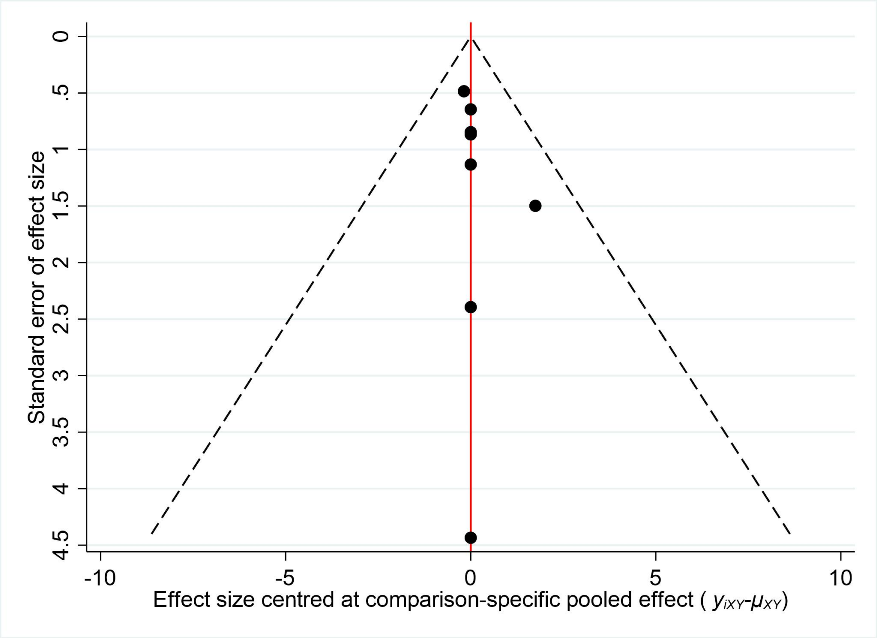

Figure S9 The funnel plot of WOMAC joint function score

Table S4 Network Meta-analysis of WOMAC Joint Function Score

Interventions	Silver needle + massage	Warm needling + massage	Common acupuncture + massage	Warm needle	Massage	Silver needle	Common acupuncture
Silver needle + massage	0						
Warm needling + massage	-3.06 (-6.00,-0.13)[1)]	0					
Common acupuncture + massage	-7.40 (-10.76,-4.04)[1)]	-4.34 (-7.79,-0.88)[1)]	0				
Warm needle	-11.03 (-20.34,-1.73)[1)]	-7.97 (-16.80,0.86)	-3.63 (-13.11,5.84)	0			
Massage	-10.40 (-12.40,-8.40)[1)]	-7.34 (-9.48,-5.19)[1)]	-3.00 (-5.71,-0.29)[1)]	0.63 (-8.45,9.72)	0		
Silver needle	-10.50 (-12.77,-8.23)[1)]	-7.44 (-10.58,-4.29)[1)]	-3.10 (-6.65,0.45)	0.53 (-8.84,9.91)	-0.10 (-2.40,2.20)	0	
Common acupuncture	-23.34 (-29.32,-17.36)[1)]	-20.28 (-26.30,-14.25)[1)]	-15.94 (-20.88,-11.00)[1)]	-12.31 (-22.99,-1.62)[1)]	-12.94 (-18.57,-7.31)[1)]	-12.84 (-18.92,-6.76)[1)]	0

Note: The difference between the two groups had statistical significance [1)] $P < 0.05$.

Above is the WOMAC joint function score

The results of sensitivity Analyses

The results of the sensitivity analysis of excluded non-conventional massage

Clinical effective rate

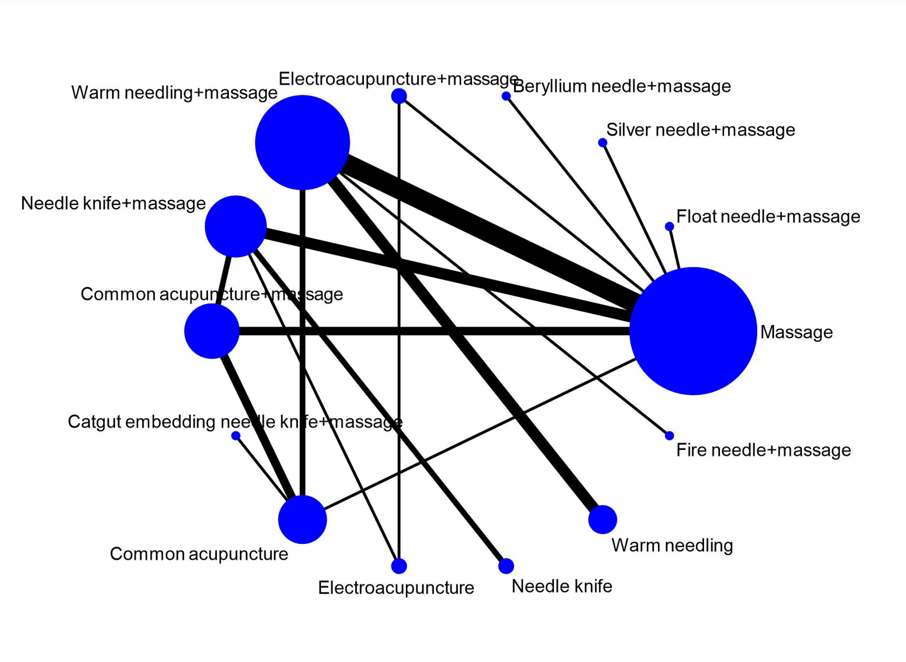

Figure S10 Network relationship diagram of clinical effective rate

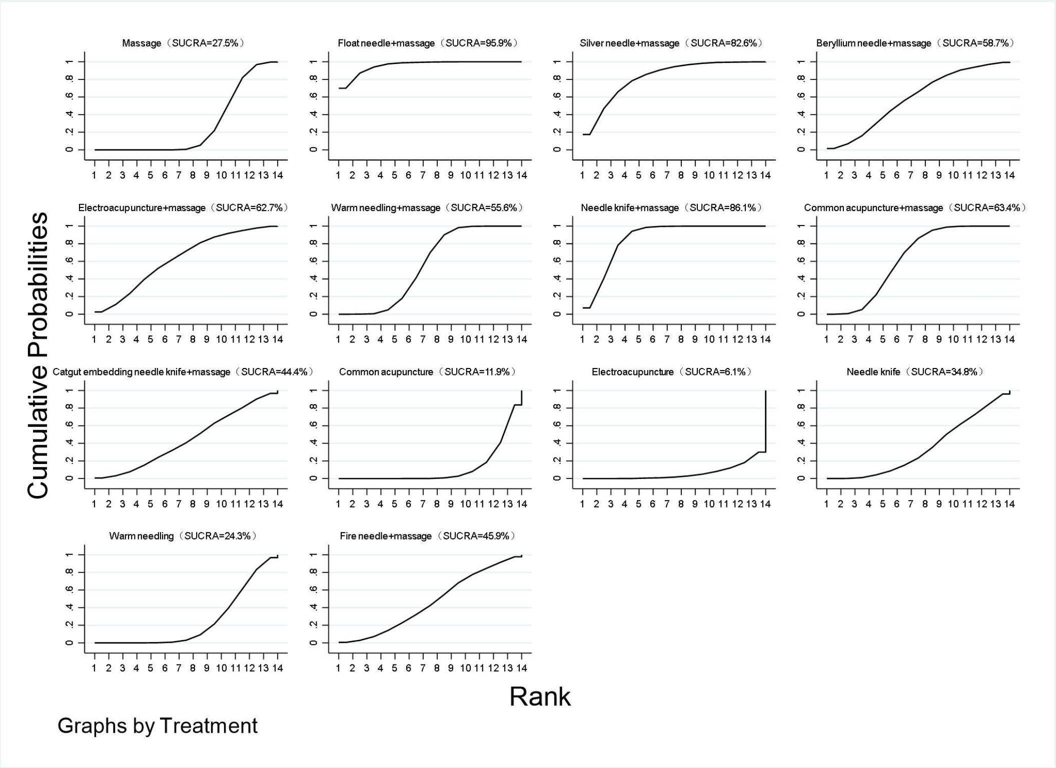

Figure S11 Sucra of clinical effective rate

Table S5 Network Meta-analysis of clinical effective rate

Interventions	Float needle + massage	Needle knife + massage	Silver needle + massage	Common acupuncture + massage	Electroacupuncture + massage	Beryllium needle + massage	Warm needling + massage
Float needle + massage	0						
Needle knife + massage	2.51 (0.46,13.78)	0					
Silver needle + massage	2.46 (0.27,22.44)	0.98 (0.17,5.51)	0				
Common acupuncture + massage	5.84 (1.08,31.63)[1)]	2.32 (1.07,5.04)[1)]	2.37 (0.43,13.23)	0			
Electroacupuncture + massage	5.56 (0.60,51.14)	2.21 (0.41,11.88)	2.26 (0.24,21.25)	0.95 (0.17,5.25)	0		
Beryllium needle + massage	6.27 (0.76,52.06)	2.50 (0.50,12.43)	2.55 (0.30,21.65)	1.07 (0.22,5.28)	1.13 (0.13,9.66)	0	
Warm needling + massage	7.35 (1.46,36.88)[1)]	2.92 (1.28,6.67)[1)]	2.99 (0.58,15.44)	1.26 (0.57,2.78)	1.32 (0.25,6.87)	1.17 (0.26,5.30)	0
Fire needle + massage	9.53 (1.09,83.04)[1)]	3.79 (0.72,20.00)	3.88 (0.44,34.52)	1.63 (0.31,8.47)	1.71 (0.19,15.33)	1.52 (0.19,12.28)	1.30 (0.31,5.50)
Catgut embedding	10.48 (1.06,103.89)[1)]	4.17 (0.69,25.10)	4.26 (0.42,43.14)	1.80 (0.32,10.05)	1.89 (0.19,19.10)	1.67 (0.18,15.43)	1.43 (0.26,7.89)

needle knife + massage							
Needle knife	13.81 (1.76,108.01)[1)]	5.49 (1.73,17.46)[1)]	5.62 (0.70,44.95)	2.37 (0.59,9.51)	2.48 (0.32,19.13)	2.20 (0.30,15.90)	1.88 (0.45,7.81)
Massage	16.00 (3.40,75.30)[1)]	6.37 (3.15,12.89)[1)]	6.51 (1.34,31.57)[1)]	2.74 (1.39,5.39)[1)]	2.88 (0.59,14.12)	2.55 (0.60,10.78)	2.18 (1.39,3.42)[1)]
Warm needling	17.64 (3.17,98.30)[1)]	7.02 (2.54,19.43)[1)]	7.18 (1.25,41.09)[1)]	3.02 (1.12,8.13)[1)]	3.17 (0.55,18.34)	2.81 (0.56,14.23)	2.40 (1.24,4.65)[1)]
Common acupuncture	27.94 (4.81,162.44)[1)]	11.12 (3.97,31.11)[1)]	11.37 (1.90,67.87)[1)]	4.79 (1.95,11.73)[1)]	5.03 (0.84,30.06)	4.45 (0.84,23.58)	3.80 (1.59,9.11)[1)]
Electroacupuncture	56.50 (5.07,629.79)[1)]	22.48 (3.77,134.18)[1)]	22.99 (2.02,261.26)[1)]	9.68 (1.45,64.81)[1)]	10.17 (1.35,76.78)[1)]	9.01 (0.86,93.84)	7.69 (1.15,51.39)[1)]

Table continued

Interventions	Fire needle + massage	Catgut embedding needle knife + massage	Needle knife	Massage	Warm needling	Common acupuncture	Electroacupuncture
Float needle + massage							
Needle knife + massage							
Silver needle + massage							
Common acupuncture + massage							
Electroacupuncture + massage							
Beryllium needle + massage							
Warm needling + massage							
Fire needle + massage	0						
Catgut embedding needle knife + massage	1.10 (0.12,10.32)	0					
Needle knife	1.45 (0.19,11.02)	1.32 (0.16,11.15)	0				
Massage	1.68 (0.37,7.63)	1.53 (0.28,8.30)	1.16 (0.30,4.49)	0			
Warm needling	1.85 (0.38,9.06)	1.68 (0.27,10.35)	1.28 (0.27,5.97)	1.10 (0.52,2.32)	0		
Common acupuncture	2.93 (0.54,15.86)	2.67 (0.61,11.61)	2.02 (0.43,9.52)	1.75 (0.76,4.03)	1.58 (0.55,4.59)	0	
Electroacupuncture	5.93 (0.55,64.49)	5.39 (0.45,64.78)	4.09 (0.49,34.35)	3.53 (0.56,22.41)	3.20 (0.44,23.42)	2.02 (0.27,15.00)	0

Note: The difference between the two groups had statistical significance [1)] $P < 0.05$.

Visual Analogue Scale

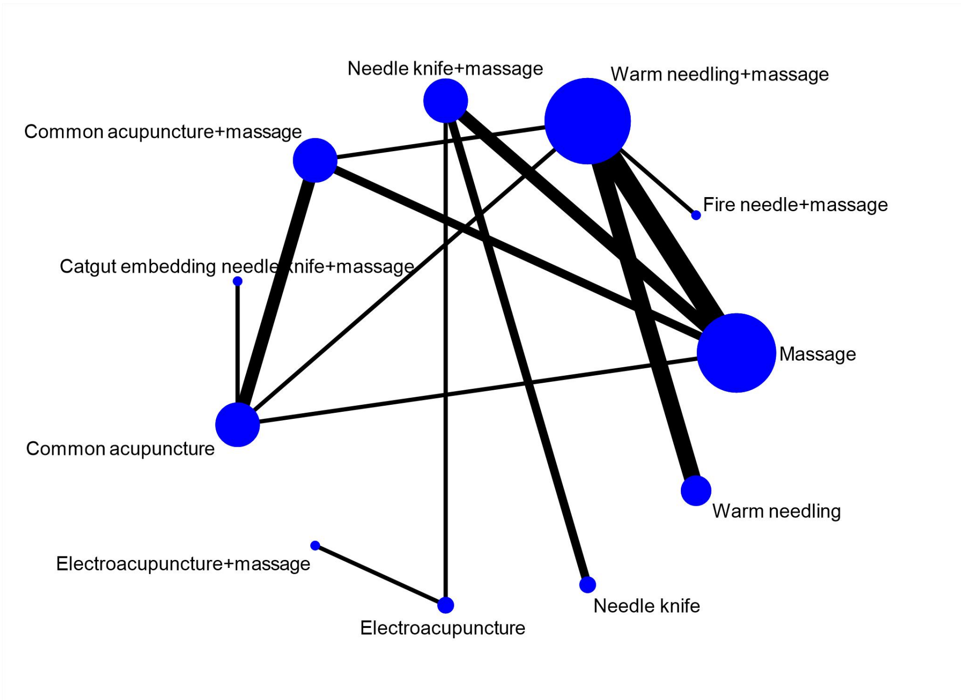

Figure S12 Network relationship diagram of VAS

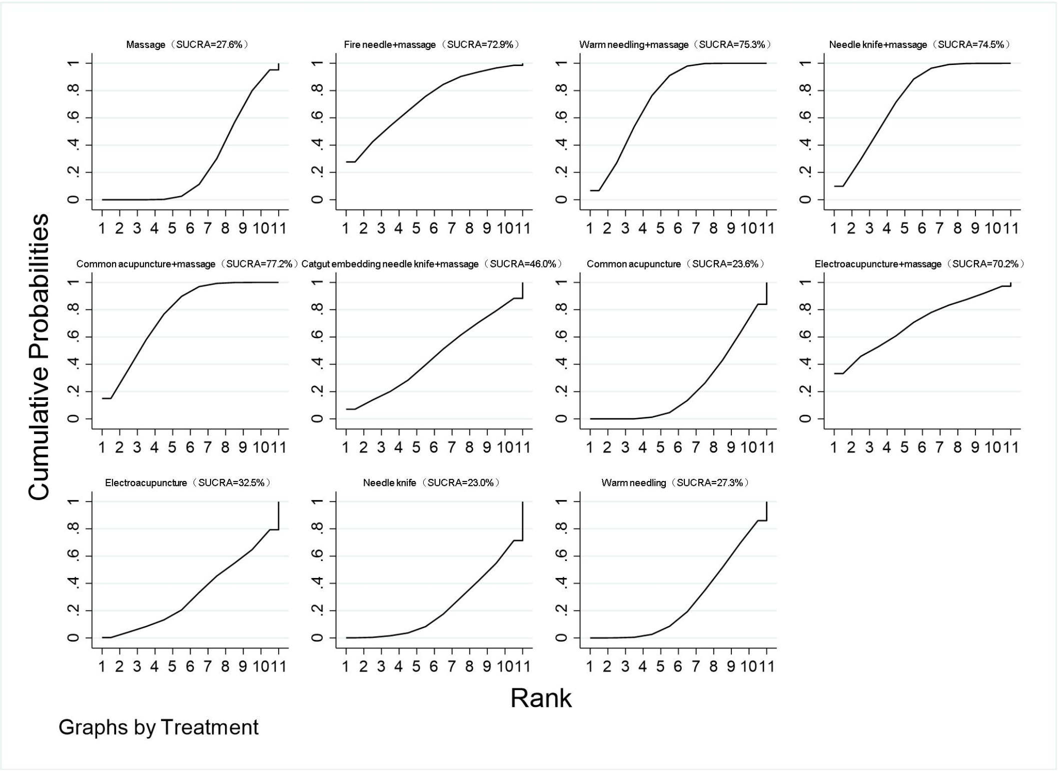

Figure S13 Sucra of VAS

Table S6 Network Meta-analysis of VAS

Interventions	Common acupuncture + massage	Warm needling + massage	Needle knife + massage	Fire needle + massage	Electroacupuncture + massage
Common acupuncture + massage	0				
Warm needling + massage	-0.09 (-1.30,1.13)	0			
Needle knife + massage	-0.13 (-1.86,1.60)	-0.04 (-1.56,1.47)	0		
Fire needle + massage	0.02 (-2.54,2.59)	0.11 (-2.15,2.37)	0.15 (-2.56,2.87)	0	
Electroacupuncture + massage	-0.05 (-3.57,3.46)	0.03 (-3.38,3.45)	0.08 (-2.99,3.14)	-0.08 (-4.17,4.02)	0
Catgut embedding needle knife + massage	-1.14 (-3.66,1.38)	-1.05 (-3.66,1.55)	-1.01 (-3.90,1.88)	-1.16 (-4.61,2.28)	-1.09 (-5.30,3.12)
Electroacupuncture	-1.65 (-4.43,1.13)	-1.57 (-4.22,1.09)	-1.52 (-3.70,0.66)	-1.68 (-5.16,1.81)	-1.60 (-3.75,0.56)
Massage	-1.73 (-2.91,-0.56)[1)]	-1.65 (-2.48,-0.81)[1)]	-1.60 (-2.86,-0.34)[1)]	-1.75 (-4.16,0.65)	-1.68 (-4.99,1.63)
Warm needling	-1.76 (-3.37,-0.15)[1)]	-1.67 (-2.75,-0.60)[1)]	-1.63 (-3.46,0.20)	-1.78 (-4.29,0.72)	-1.71 (-5.27,1.86)
Common acupuncture	-1.88 (-3.03,-0.73)[1)]	-1.79 (-3.12,-0.47)[1)]	-1.75 (-3.58,0.08)	-1.90 (-4.52,0.71)	-1.83 (-5.39,1.74)
Needle knife	-1.99 (-4.29,0.30)	-1.91 (-4.04,0.23)	-1.86 (-3.37,-0.35)[1)]	-2.02 (-5.13,1.09)	-1.94 (-5.36,1.48)

Table continued

Interventions	Catgut embedding needle knife + massage	Electroacupuncture	Massage	Warm needling	Common acupuncture	Needle knife
Common acupuncture + massage						
Warm needling + massage						
Needle knife + massage						
Fire needle + massage						
Electroacupuncture + massage						
Catgut embedding needle knife + massage	0					
Electroacupuncture	-0.51 (-4.13,3.11)	0				

Massage	-0.59 (-3.19,2.01)	-0.08 (-2.60,2.44)	0			
Warm needling	-0.62 (-3.43,2.18)	-0.11 (-2.95,2.73)	-0.03 (-1.35,1.29)	0		
Common acupuncture	-0.74 (-2.98,1.50)	-0.23 (-3.07,2.61)	-0.15 (-1.47,1.17)	-0.12 (-1.81,1.57)	0	
Needle knife	-0.85 (-4.11,2.41)	-0.34 (-2.99,2.31)	-0.26 (-2.23,1.71)	-0.23 (-2.60,2.14)	-0.11 (-2.48,2.26)	0

Note: The difference between the two groups had statistical significance [1)] $P < 0.05$.

Total Lysholm Score

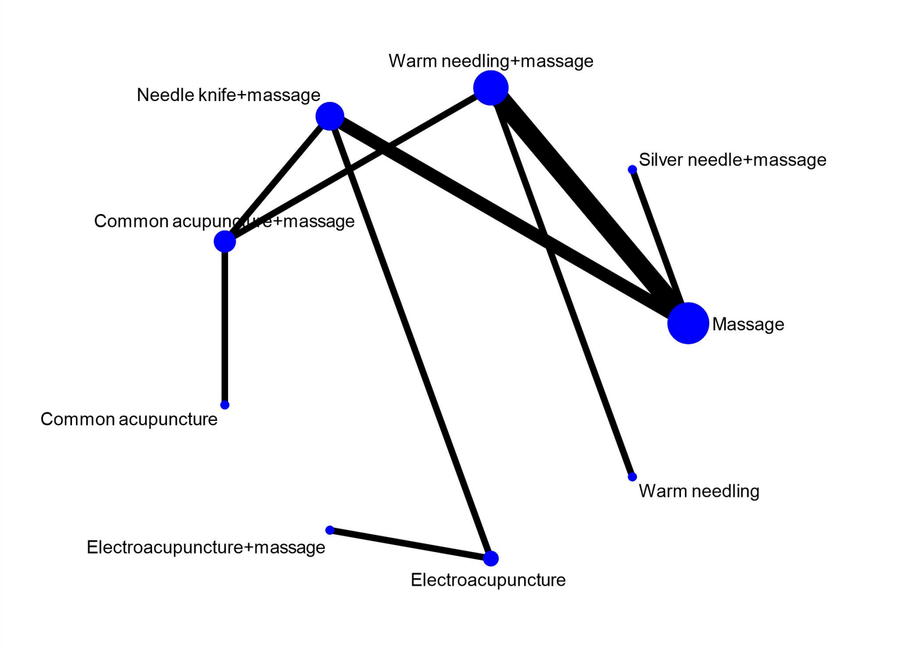

Figure S14 Network relationship diagram of Total lysholm score

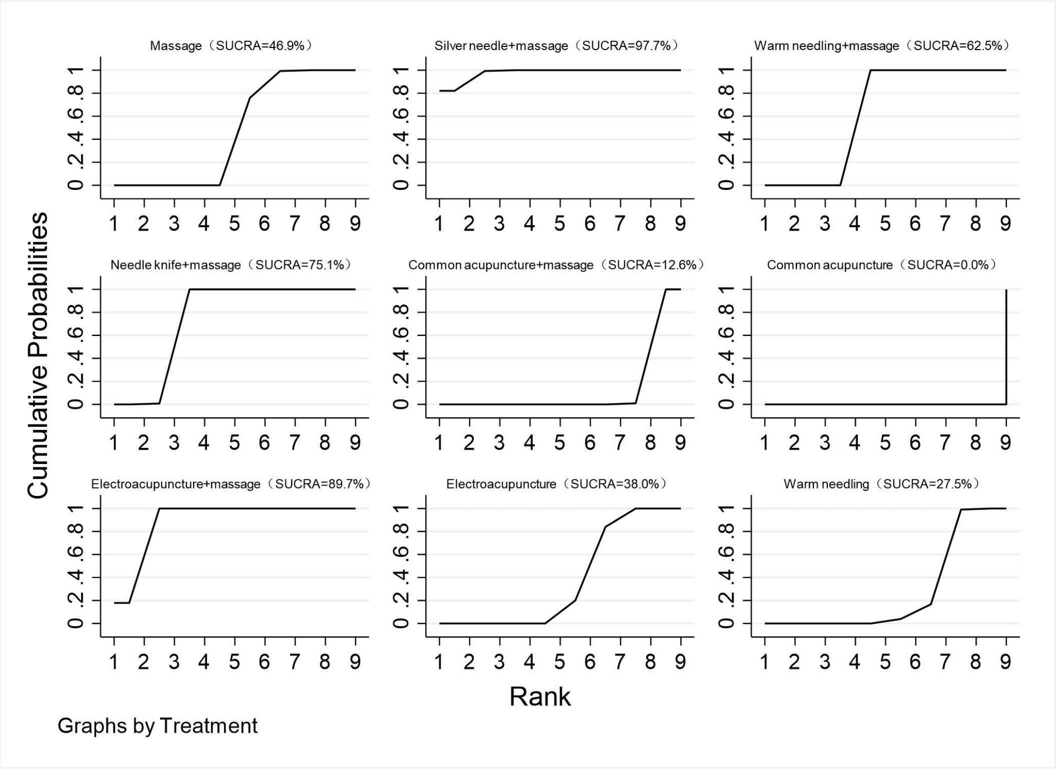

Figure S15 Sucra of Total lysholm score

Table S7 Network Meta-analysis of Total Lysholm Score

Interventions	Silver needle + massage	Electroacupuncture + massage	Needle knife + massage	Warm needling + massage	Massage
Silver needle + massage	0				
Electroacupuncture + massage	2.69 (-3.19,8.57)	0			
Needle knife + massage	7.15 (1.52,12.78)[1)]	4.46 (2.77,6.15)[1)]	0		
Warm needling + massage	13.10 (7.74,18.46)[1)]	10.41 (7.81,13.02)[1)]	5.95 (3.97,7.94)[1)]	0	
Massage	18.81 (13.51,24.11)[1)]	16.12 (13.58,18.67)[1)]	11.66 (9.76,13.57)[1)]	5.71 (4.91,6.51)[1)]	0
Electroacupuncture	19.69 (13.99,25.39)[1)]	17.00 (15.57,18.43)[1)]	12.54 (11.65,13.43)[1)]	6.59 (4.41,8.76)[1)]	0.88 (-1.22,2.98)[1)]
Warm needling	21.63 (15.45,27.82)[1)]	18.94 (14.90,22.98)[1)]	14.48 (10.81,18.16)[1)]	8.53 (5.44,11.62)[1)]	2.82 (-0.37,6.01)[1)]
Common acupuncture + massage + massage	25.92 (20.26,31.58)[1)]	23.23 (20.46,26.01)[1)]	18.77 (16.57,20.98)[1)]	12.82 (10.91,14.73)[1)]	7.11 (5.12,9.10)[1)]
Common acupuncture + massage	44.13 (37.06,51.19)[1)]	41.44 (36.38,46.50)[1)]	36.98 (32.21,41.75)[1)]	31.03 (26.39,35.67)[1)]	25.32 (20.65,29.99)[1)]

Table continued

Interventions	Electroacupuncture	Warm needling	Common acupuncture + massage + massage	Common acupuncture
Silver needle + massage				
Electroacupuncture + massage				
Needle knife + massage				
Warm needling + massage				
Massage				
Electroacupuncture	0			
Warm needling	1.94 (-1.83,5.72)	0		
Common acupuncture + massage + massage	6.23 (3.86,8.61)[1)]	4.29 (0.66,7.92)[1)]	0	
Common acupuncture	24.44 (19.59,29.29)[1)]	22.50 (16.92,28.07)[1)]	18.21 (13.98,22.44)[1)]	0

Note: The difference between the two groups had statistical significance [1)] $P < 0.05$.

The results sensitivity Analyses of Risk of bias

Clinical effective rate

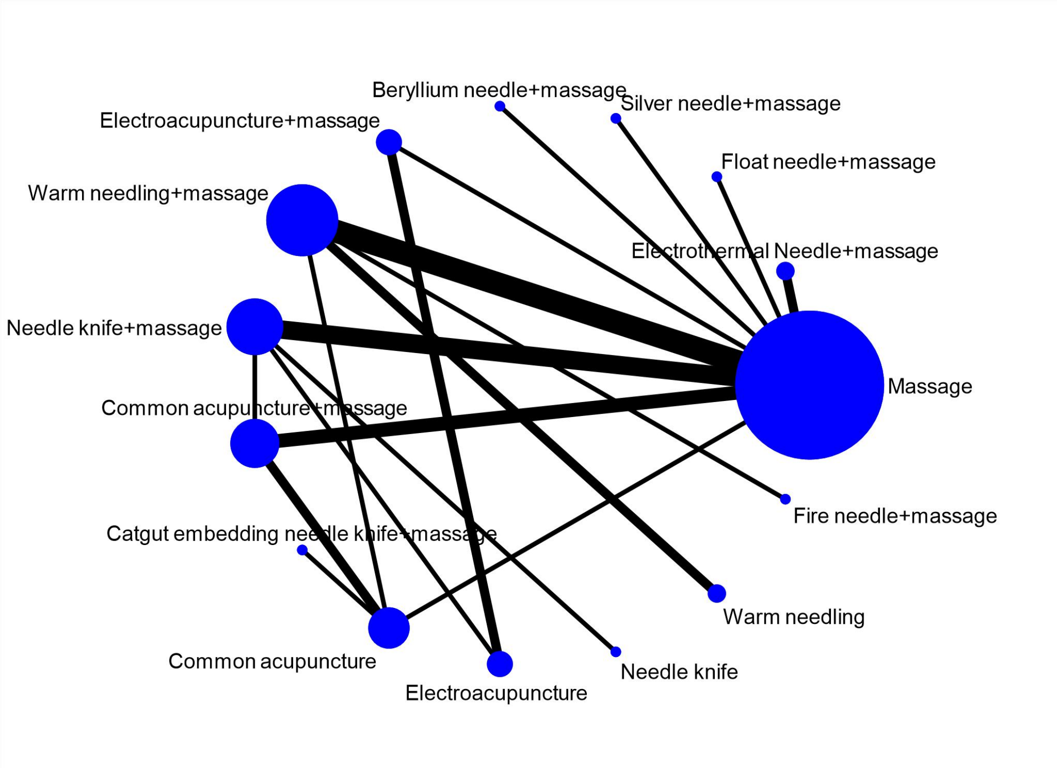

Figure S16 Network relationship diagram of Clinical effective rate

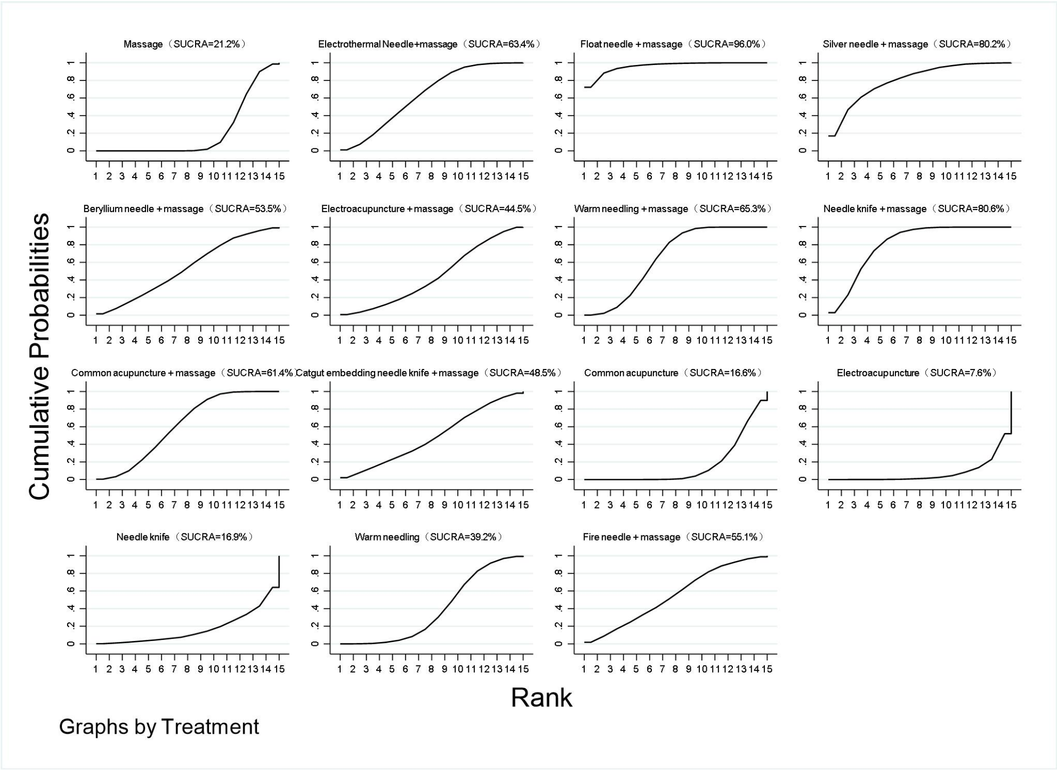

Figure S17 Sucra of Clinical effective rate

Table S8 Network Meta-analysis of clinical effective rate

Interventions	Float needle + massage	Needle knife + massage	Silver needle + massage	Warm needling + massage	Electrothermal needle + massage	Common acupuncture + massage	Fire needle + massage
Float needle + massage	0						
Needle knife + massage	3.02 (0.57,15.98)	0					
Silver needle + massage	2.46 (0.28,21.66)	0.81 (0.15,4.42)	0				
Warm needling + massage	4.69 (0.92,23.95)	1.55 (0.64,3.76)	1.91 (0.36,10.02)	0			
Electrothermal needle + massage	4.84 (0.79,29.81)	1.60 (0.48,5.31)	1.97 (0.31,12.45)	1.03 (0.33,3.26)	0		
Common acupuncture + massage	5.05 (0.88,28.97)	1.67 (0.63,4.43)	2.06 (0.35,12.11)	1.08 (0.39,2.95)	1.04 (0.28,3.86)	0	
Fire needle + massage	6.07 (0.70,52.71)	2.01 (0.38,10.69)	2.47 (0.28,21.92)	1.30 (0.31,5.35)	1.25 (0.20,7.78)	1.20 (0.21,6.84)	0
Beryllium needle + massage	6.27 (0.78,50.15)	2.08 (0.43,9.94)	2.55 (0.31,20.87)	1.34 (0.29,6.18)	1.30 (0.23,7.29)	1.24 (0.24,6.48)	1.03 (0.13,8.31)
Catgut embedding needle knife + massage	7.45 (0.74,74.70)	2.46 (0.39,15.44)	3.03 (0.30,31.01)	1.59 (0.27,9.29)	1.54 (0.21,11.30)	1.47 (0.24,8.89)	1.23 (0.13,11.80)
Electroacupuncture + massage	8.45 (1.04,68.62)[1)]	2.80 (0.63,12.38)	3.44 (0.41,28.55)	1.80 (0.38,8.50)	1.74 (0.30,10.00)	1.67 (0.32,8.70)	1.39 (0.17,11.36)
Warm needle	9.30 (1.56,55.56)[1)]	3.08 (0.98,9.70)	3.78 (0.62,23.20)	1.99 (0.96,4.12)	1.92 (0.49,7.50)	1.84 (0.53,6.38)	1.53 (0.31,7.54)
Massage	16.00 (3.49,73.41)[1)]	5.29 (2.70,10.38)[1)]	6.51 (1.38,30.79)[1)]	3.41 (1.91,6.12)[1)]	3.30 (1.23,8.90)[1)]	3.17 (1.35,7.45)[1)]	2.63 (0.57,12.20)
Needle knife	28.11 (1.90,416.11)[1)]	9.30 (1.12,77.37)[1)]	11.44 (0.76,172.27)	6.00 (0.60,59.61)	5.81 (0.51,66.19)	5.57 (0.54,57.30)	4.63 (0.31,68.74)
Common	19.86 (3.29,119.79)[1)]	6.57 (2.12,20.38)[1)]	8.08 (1.31,50.02)[1)]	4.24	4.10	3.93	3.27 (0.57,18.70)

acupuncture				(1.53,11.71)[1)]	(1.04,16.22)[1)]	(1.35,11.45)[1)]	
Electroacupuncture	37.88 (4.34,330.44)[1)]	12.53 (2.71,58.00)[1)]	15.41 (1.73,137.38)[1)]	8.09 (1.56,41.89)[1)]	7.82 (1.25,48.82)[1)]	7.50 (1.34,42.12)[1)]	6.24 (0.71,54.69)

Interventions	Beryllium needle + massage	Catgut embedding needle knife + massage	Electroacupuncture + massage	Warm needle	Massage	Needle knife	Common acupuncture	Electroacupuncture
Float needle + massage								
Needle knife + massage								
Silver needle + massage								
Warm needling + massage								
Electrothermal needle + massage								
Common acupuncture + massage								
Fire needle + massage								
Beryllium needle + massage	0							
Catgut embedding needle knife + massage	1.19 (0.13,11.09)	0						
Electroacupuncture + massage	1.35 (0.18,10.12)	1.13 (0.12,10.70)	0					
Warm needle	1.48 (0.27,8.08)	1.25 (0.18,8.44)	1.10 (0.20,6.11)	0				

Massage	2.55 (0.62,10.49)	2.15 (0.38,12.13)	1.89 (0.45,7.97)	1.72 (0.68,4.38)	0				
Needle knife	4.48 (0.32,62.46)	3.78 (0.23,62.26)	3.33 (0.25,44.30)	3.02 (0.27,33.62)	1.76 (0.19,16.22)	0			
Common acupuncture	3.17 (0.58,17.42)	2.67 (0.63,11.31)	2.35 (0.42,13.09)	2.13 (0.61,7.46)	1.24 (0.48,3.22)	0.71 (0.06,7.80)	0		
Electroacupuncture	6.04 (0.75,48.85)	5.09 (0.51,51.11)	4.48 (1.38,14.58)[1)]	4.07 (0.67,24.63)	2.37 (0.51,11.04)	1.35 (0.10,18.40)	1.91 (0.32,11.53)	0	

Table continued

Note: The difference between the two groups had statistical significance [1)] $P < 0.05$.

Visual Analogue Scale

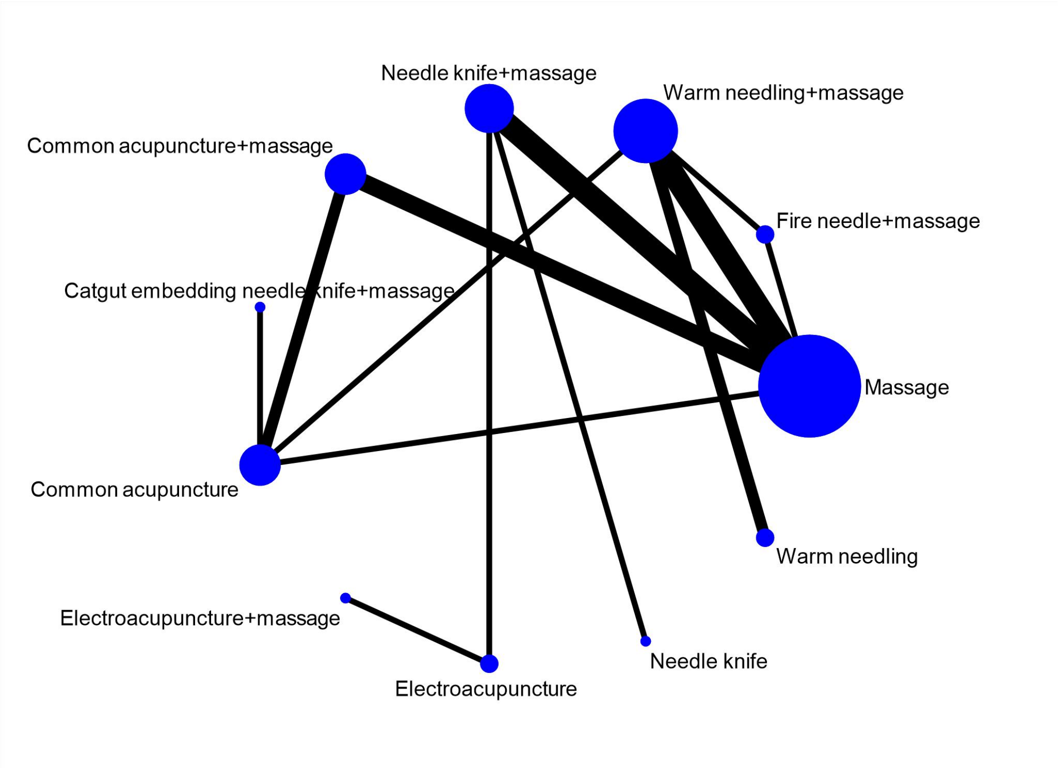

Figure S18 Network relationship diagram of VAS

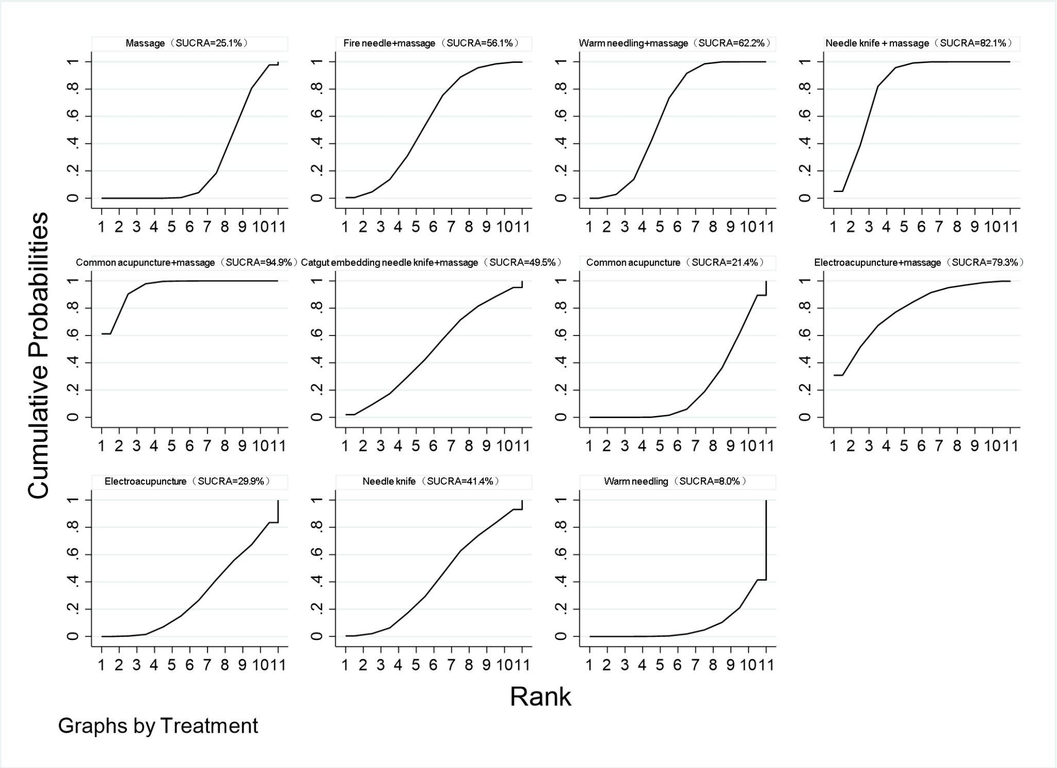

Figure S19 Sucra of VAS

Table S9 Network Meta-analysis of VAS

Interventions	Common acupuncture + massage	Needle knife + massage	Electroacupuncture + massage	Warm needling + massage	Fire needle + massage	Catgut embedding needle knife + massage
Common acupuncture + massage	0					
Needle knife + massage	-0.60 (-1.57,0.38)	0				
Electroacupuncture + massage	-0.52 (-2.57,1.54)	0.08 (-1.73,1.89)	0			
Warm needling + massage	-1.22 (-2.06,-0.38)[1)]	-0.62 (-1.50,0.25)	-0.70 (-2.72,1.31)	0		
Fire needle + massage	-1.37 (-2.56,-0.17)[1)]	-0.77 (-1.97,0.43)	-0.85 (-3.03,1.32)	-0.15 (-1.15,0.85)	0	
Catgut embedding needle knife + massage	-1.56 (-3.19,0.07)	-0.96 (-2.72,0.79)	-1.04 (-3.57,1.48)	-0.34 (-1.99,1.30)	-0.19 (-2.06,1.68)	0
Needle knife	-1.77 (-3.35,-0.18)[1)]	-1.17 (-2.42,0.08)	-1.25 (-3.45,0.95)	-0.55 (-2.07,0.98)	-0.40 (-2.13,1.33)	-0.21 (-2.36,1.95)
Electroacupuncture	-2.12 (-3.74,-0.49)[1)]	-1.52 (-2.82,-0.22)[1)]	-1.60 (-2.86,-0.34)[1)]	-0.90 (-2.47,0.67)	-0.75 (-2.52,1.02)	-0.56 (-2.75,1.63)

Massage	-2.20 (-2.89,-1.50)[1)]	-1.60 (-2.28,-0.92)[1)]	-1.68 (-3.62,0.25)	-0.98 (-1.53,-0.43)[1)]	-0.83 (-1.82,0.16)	-0.64 (-2.26,0.98)
Common acupuncture	-2.30 (-3.13,-1.47)[1)]	-1.70 (-2.77,-0.64)[1)]	-1.78 (-3.88,0.32)	-1.08 (-1.94,-0.22)[1)]	-0.93 (-2.17,0.31)	-0.74 (-2.14,0.66)
Warm needling	-2.82 (-4.02,-1.61)[1)]	-2.22 (-3.45,-0.99)[1)]	-2.30 (-4.49,-0.11)[1)]	-1.60 (-2.46,-0.73)[1)]	-1.45 (-2.77,-0.12)[1)]	-1.26 (-3.12,0.60)

Interventions	Needle knife	Electroacupuncture	Massage	Common acupuncture	Warm needling
Common acupuncture + massage					
Needle knife + massage					
Electroacupuncture + massage					
Warm needling + massage					
Fire needle + massage					
Catgut embedding needle knife + massage					
Needle knife	0				
Electroacupuncture	-0.35 (-2.16,1.46)	0			
Massage	-0.43 (-1.85,0.99)	-0.08 (-1.55,1.39)	0		
Common acupuncture	-0.53 (-2.17,1.11)	-0.18 (-1.86,1.50)	-0.10 (-0.92,0.72)	0	
Warm needling	-1.05 (-2.80,0.70)	-0.70 (-2.49,1.09)	-0.62 (-1.65,0.41)	-0.52 (-1.74,0.70)	0

Table continued

Note: The difference between the two groups had statistical significance [1)] $P < 0.05$.

Total Lysholm Score

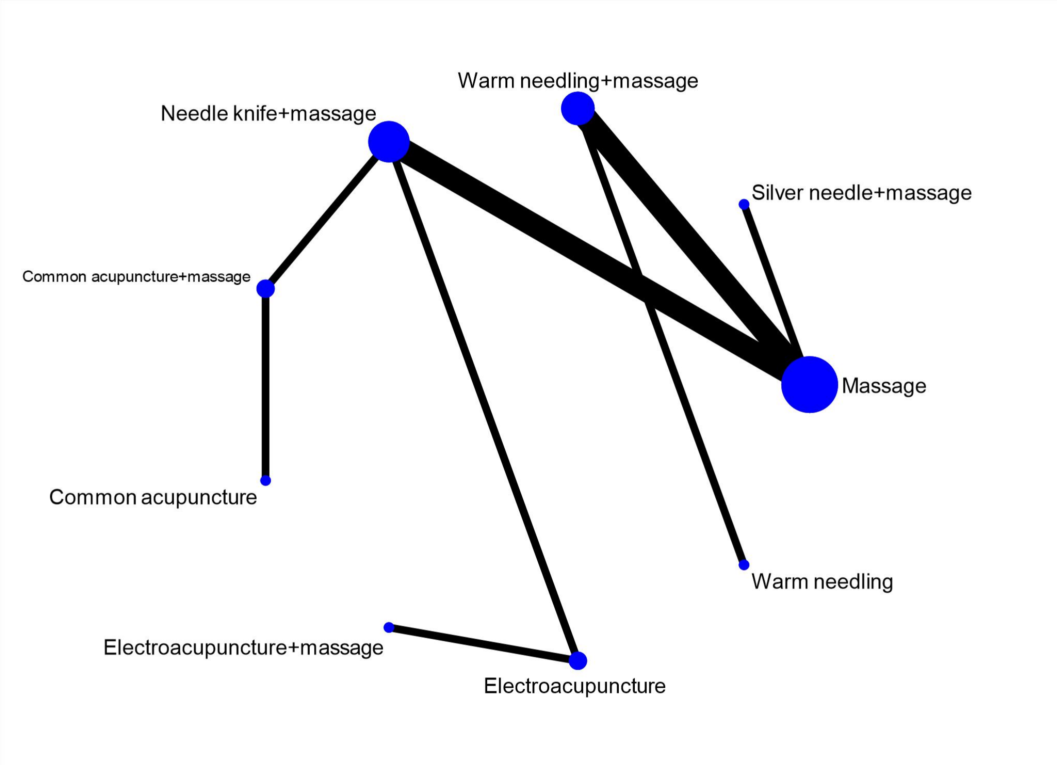

Figure S20 Network relationship diagram of Total lysholm score

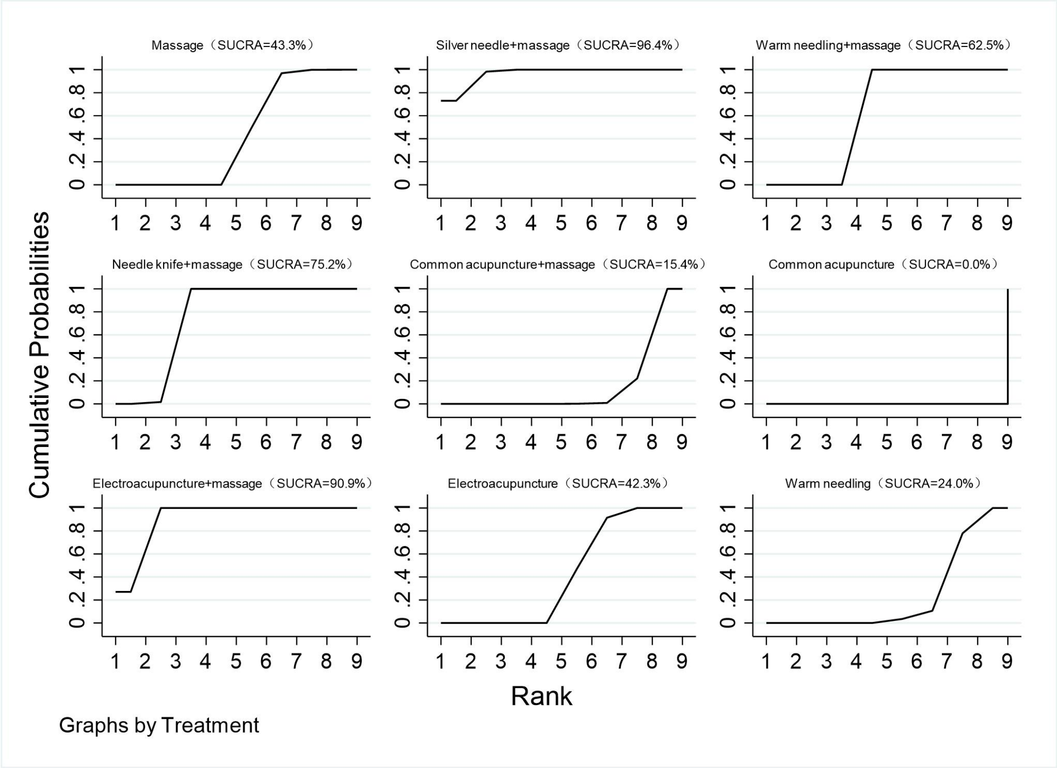

Figure S21 Sucra of Total lysholm score

Table S10 Network Meta-analysis of Total Lysholm Score

Interventions	Silver needle + massage	Electroacupuncture + massage	Needle knife + massage	Warm needling + massage	Massage
Silver needle + massage	0				
Electroacupuncture + massage	1.83 (-4.12,7.79)	0			
Needle knife + massage	6.29 (0.58,12.00)[1)]	4.46 (2.77,6.15)[1)]	0		
Warm needling + massage	13.03 (7.66,18.40)[1)]	11.20 (8.34,14.05)[1)]	6.74 (4.43,9.04)[1)]	0	
Massage	18.81 (13.51,24.11)[1)]	16.98 (14.26,19.69)[1)]	12.52 (10.39,14.65)[1)]	5.78 (4.90,6.66)[1)]	0
Electroacupuncture	18.83 (13.05,24.61)[1)]	17.00 (15.57,18.43)[1)]	12.54 (11.65,13.43)[1)]	5.80 (3.33,8.27)[1)]	0.02 (-2.28,2.33)
Warm needle	21.56 (15.36,27.76)[1)]	19.73 (15.52,23.93)[1)]	15.27 (11.41,19.12)[1)]	8.53 (5.44,11.62)[1)]	2.75 (-0.46,5.96)
Common acupuncture + massage	23.48 (17.02,29.95)[1)]	21.65 (18.18,25.12)[1)]	17.19 (14.16,20.22)[1)]	10.45 (6.65,14.26)[1)]	4.67 (0.97,8.38)[1)]
Common acupuncture	41.69 (33.97,49.42)[1)]	39.86 (34.39,45.33)[1)]	35.40 (30.20,40.60)[1)]	28.66 (22.97,34.35)[1)]	22.88 (17.26,28.50)[1)]

Table continued

Interventions	Electroacupuncture	Warm needle	Common acupuncture + massage	Common acupuncture
Silver needle + massage				
Electroacupuncture + massage				
Needle knife +				

massage				
Warm needling + massage				
Massage				
Electroacupuncture	0			
Warm needle	2.73 (-1.23,6.68)	0		
Common acupuncture + massage	4.65 (1.49,7.81)[1)]	1.92 (-2.98,6.82)	0	
Common acupuncture	22.86 (17.58,28.13)[1)]	20.13 (13.66,26.60)[1)]	18.21 (13.98,22.44)[1)]	0

Note: The difference between the two groups had statistical significance [1)] $P < 0.05$.

Subgroup analysis

Table S11 Network Meta-analysis of Clinical effective rate(<3weeks group)

Treatment	FTN+MA	NK+MA	SN+MA	CA+MA	BN+MA	WN+MA	FN+MA	MA	WN	CA
FTN+MA	0									
NK+MA	2.54 (0.35,18.54)	0								
SN+MA	2.46 (0.26,23.30)	0.97 (0.13,7.26)	0							
CA+MA	3.94 (0.61,25.40)	1.55 (0.32,7.47)	1.60 (0.24,10.60)	0						
BN+MA	6.27 (0.73,54.13)	2.47 (0.37,16.67)	2.55 (0.29,22.50)	1.59 (0.27,9.42)	0					
WN+MA	8.63 (1.63,45.61)[1)]	3.41 (0.91,12.80)	3.51 (0.65,19.08)	2.19 (0.73,6.56)	1.38 (0.29,6.58)	0				
FN+MA	11.19 (1.21,103.32)[1)]	4.41 (0.61,32.00)	4.55 (0.48,42.93)	2.84 (0.45,17.82)	1.78 (0.21,15.30)	1.30 (0.30,5.65)	0			
MA	16.00 (3.31,77.33)[1)]	6.31 (1.87,21.27)[1)]	6.51 (1.31,32.41)[1)]	4.06 (1.50,11.01)[1)]	2.55 (0.59,11.10)	1.85 (1.08,3.17)[1)]	1.43 (0.30,6.85)	0		
WN	21.69 (3.58,131.27)[1)]	8.56 (1.91,38.23)[1)]	8.83 (1.42,54.81)[1)]	5.51 (1.49,20.34)[1)]	3.46 (0.63,19.10)	2.51 (1.11,5.71)[1)]	1.94 (0.36,10.46)	1.36 (0.57,3.24)	0	
CA	30.70 (3.58,263.10)[1)]	12.11 (1.81,80.84)[1)]	12.49 (1.43,109.40)[1)]	7.80 (2.04,29.75)[1)]	4.89 (0.62,38.87)	3.56 (0.82,15.49)	2.74 (0.34,22.01)	1.92 (0.45,8.27)	1.42 (0.27,7.39)	0

Note:FTN=floating needle；NK=needle knife；SN=silver needle；CA=common acupuncture；BN=beryllium needle；WN=warming needle；FN=fire needle；MA；massage

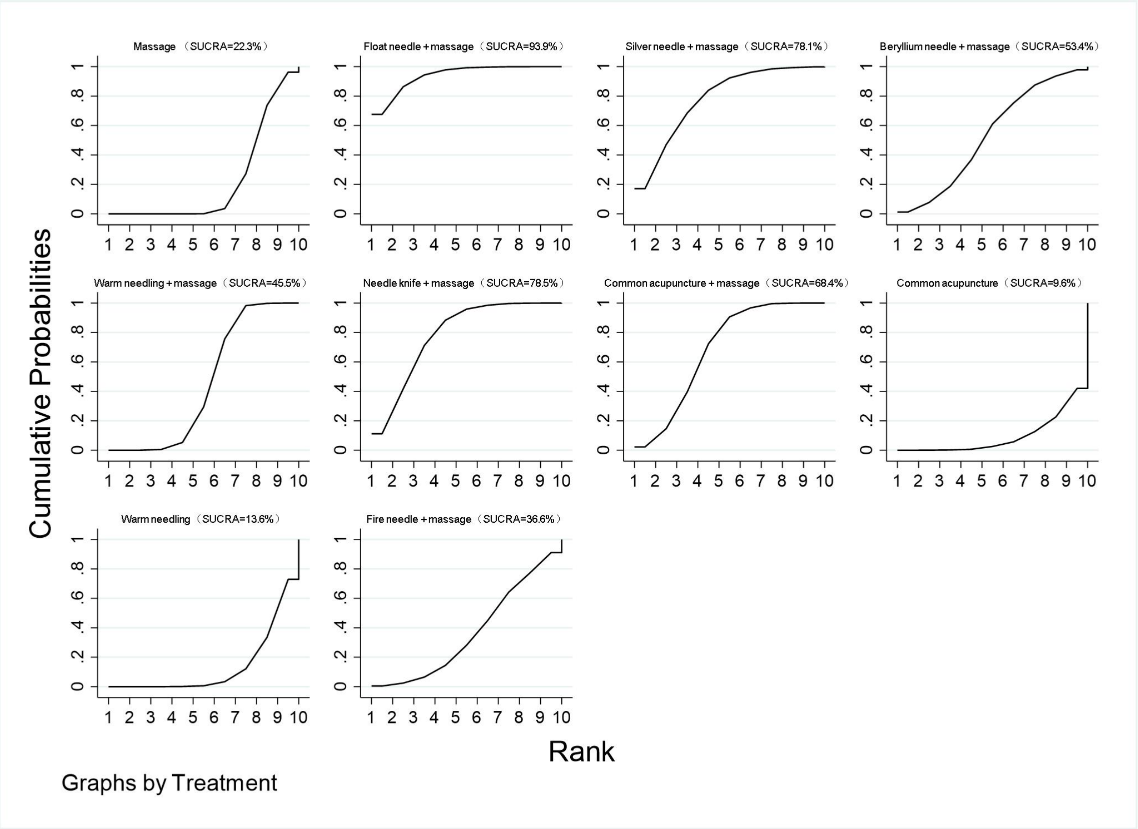

Figure S22 Sucra of Clinical effective rate(<3weeks group)

Table S12 Network Meta-analysis of Clinical effective rate(≥3weeks group)

Treatment	NK+MA	WN+MA	ETN+MA	CENK+MA	CA+MA	EA+MA	WN	MA	NK	CA	EA
NK+MA	0										
WN+MA	1.44 (0.51,4.10)	0									
ETN+MA	1.50 (0.44,5.14)	1.04 (0.30,3.62)	0								
CENK+MA	2.17 (0.36,12.98)	1.50 (0.22,10.36)	1.44 (0.19,11.04)	0							
CA+MA	2.40 (1.05,5.47)	1.66 (0.52,5.31)	1.59 (0.42,6.02)	1.11 (0.17,7.03)	0						
EA+MA	2.68 (0.60,12.00)	1.86 (0.37,9.43)	1.78 (0.31,10.24)	1.24 (0.13,11.79)	1.12 (0.22,5.70)	0					
WN	3.14 (0.51,19.48)	2.17 (0.49,9.72)	2.09 (0.30,14.61)	1.45 (0.13,16.71)	1.31 (0.20,8.72)	1.17 (0.13,10.67)	0				

MA	4.97 (2.41,10.27)[1)]	3.44 (1.62,7.30)[1)]	3.30 (1.23,8.90)[1)]	2.30 (0.39,13.62)	2.07 (0.85,5.04)	1.85 (0.44,7.83)	1.58 (0.30, 8.46)	0			
NK	5.47 (1.76,17.04)[1)]	3.79 (0.81,17.73)	3.64 (0.68,19.37)	2.53 (0.30,21.06)	2.28 (0.56,9.29)	2.04 (0.31,13.38)	1.74 (0.20, 14.96)	1.10 (0.29,4.24)	0		
CA	5.78 (2.01,16.63)[1)]	4.00 (1.11,14.43)[1)]	3.84 (0.91,16.15)	2.67 (0.63,11.31)	2.41 (0.76,7.64)	2.15 (0.38,12.14)	1.84 (0.26, 13.22)	1.16 (0.41,3.29)	1.06 (0.22,4.98)	0	
EA	12.17 (2.62,56.60)[1)]	8.43 (1.51,47.01)[1)]	8.09 (1.29,50.73)[1)]	5.62 (0.56,56.19)	5.08 (0.94,27.42)	4.54 (1.39,14.77)[1)]	3.88 (0.40, 37.88)	2.45 (0.52,11.48)	2.22 (0.33,15.03)	2.11 (0.35,12.66)	0

Note:NK=needle knife；WN=warming needle；ETN=electrothermal needle；CENK=catgut embedding needle knife；CA=common acupuncture；EA=electroacupuncture；MA=massage

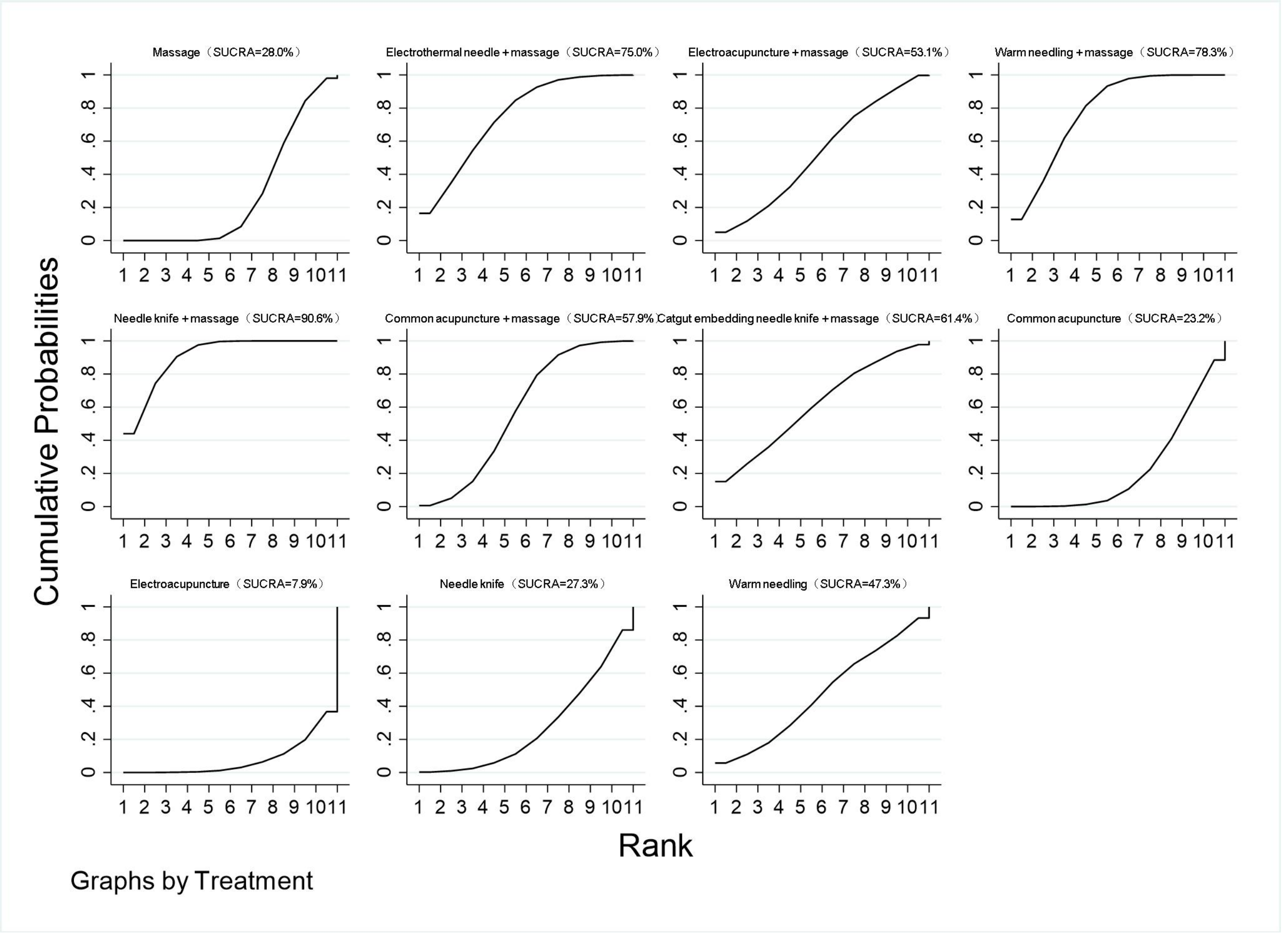

Figure S23 Sucra of Clinical effective rate(≥3weeks group)

Visual Analogue Scale(<3weeks group)

Table S13 Network Meta-analysis of VAS(<3weeks group)

Treatment	CA+MA	NK+MA	WN+MA	FN+MA	CA	MA	WN
CA+MA	0						
NK+MA	-0.16 (-2.73,2.41)	0					
WN+MA	-0.53 (-2.84,1.79)	-0.37 (-2.05,1.31)	0				
FN+MA	-0.86 (-3.49,1.76)	-0.70 (-2.79,1.38)	-0.34 (-1.87,1.20)	0			
CA	-1.82 (-3.91,0.27)	-1.66 (-4.97,1.65)	-1.29 (-4.41,1.82)	-0.96 (-4.31,2.40)	0		
MA	-1.89 (-4.03,0.25)	-1.73 (-3.16,-0.30)[1)]	-1.36 (-2.25,-0.47)[1)]	-1.03 (-2.55,0.50)	-0.07 (-3.06,2.92)	0	
WN	-2.29 (-4.83,0.26)	-2.13 (-4.12,-0.14)[1)]	-1.76 (-2.88,-0.64)[1)]	-1.42 (-3.31,0.46)	-0.46 (-3.76,2.83)	-0.40 (-1.78,0.99)	0

Note:CA=common acupuncture； NK=needle knife； WN=warming needle； FN=fire needle； MA=massage

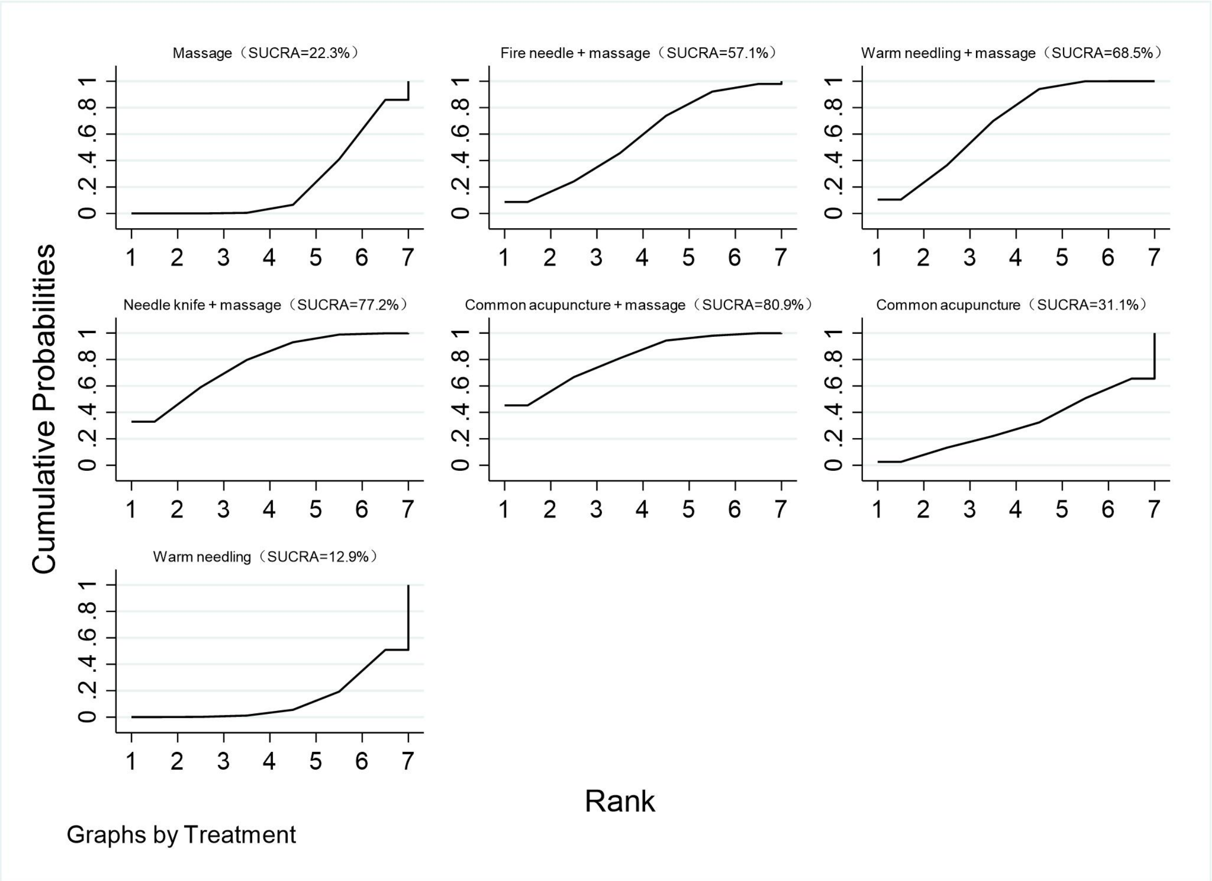

Figure S24 Sucra of VAS(<3weeks group)

Visual Analogue Scale(⩾3weeks group)

Table S14 Network Meta-analysis of VAS(≥3weeks group)

Treatment	CA+MA	WN+MA	NK+MA	EA+MA	CENK+MA	WN	EA	MA	CA	NK
CA+MA	0									
WN+MA	-0.09 (-1.66,1.49)	0								
NK+MA	-0.37 (-2.71,1.98)	-0.28 (-2.61,2.05)	0							
EA+MA	-0.29 (-4.54,3.96)	-0.20 (-4.44,4.03)	0.08 (-3.47,3.62)	0						
CENK+MA	-1.25 (-4.26,1.75)	-1.17 (-4.24,1.91)	-0.89 (-4.47,2.70)	-0.96 (-6.00,4.07)	0					
WN	-1.32 (-4.44,1.81)	-1.23 (-3.93,1.47)	-0.95 (-4.52,2.62)	-1.03 (-6.05,4.00)	-0.06 (-4.16,4.03)	0				
EA	-1.89 (-5.33,1.55)	-1.80 (-5.23,1.62)	-1.52 (-4.04,1.00)	-1.60 (-4.10,0.90)	-0.64 (-5.01,3.74)	-0.57 (-4.93,3.79)	0			
MA	-1.80 (-3.27,-0.34)[1)]	-1.72 (-3.15,-0.29)[1)]	-1.44 (-3.27,0.40)	-1.52 (-5.50,2.47)	-0.55 (-3.63,2.52)	-0.49 (-3.55,2.57)	0.08 (-3.03,3.19)	0		
CA	-1.99 (-3.55,-0.44)[1)]	-1.91 (-3.59,-0.22)[1)]	-1.63 (-4.12,0.87)	-1.70 (-6.04,2.63)	-0.74 (-3.31,1.83)	-0.67 (-3.86,2.51)	-0.10 (-3.65,3.44)	-0.19 (-1.88,1.51)	0	
NK	-2.23 (-5.15,0.70)	-2.14 (-5.05,0.77)	-1.86 (-3.62,-0.10)[1)]	-1.94 (-5.89,2.02)	-0.97 (-4.96,3.02)	-0.91 (-4.88,3.06)	-0.34 (-3.41,2.73)	-0.42 (-2.96,2.12)	-0.23 (-3.29,2.82)	0

Note:CA=common acupuncture；WN=warming needle；NK=needle knife；EA=electroacupuncture；CENK=catgut embedding needle knife；MA；massage

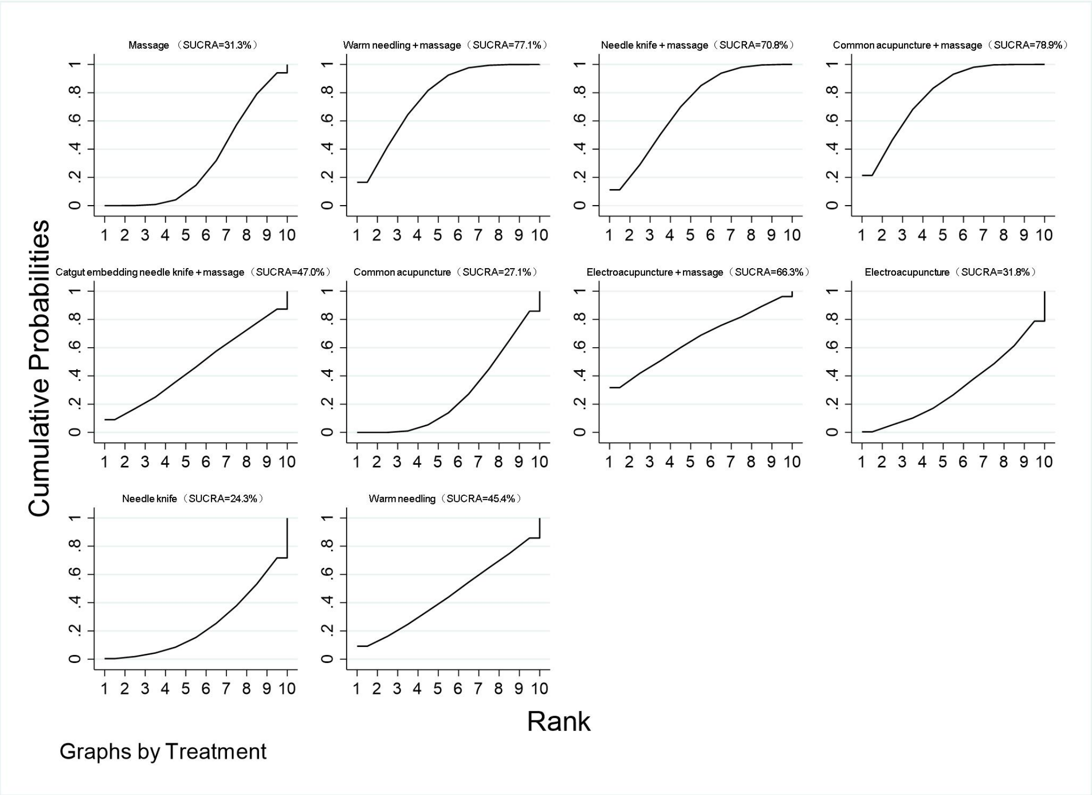

Figure S25 Sucra of VAS(≥3weeks group)

Total Lysholm Score(<3weeks group)

Table S15 Network Meta-analysis of Total lysholm score(<3weeks group)

Treatment	SN+MA	NK+MA	CA+MA	WN+MA	MA	CA	WN
SN+MA	0						
NK+MA	6.31 (0.58,12.05)[1)]	0					
CA+MA	10.81 (5.28,16.34)[1)]	4.50 (1.78,7.21)[1)]	0				
WN+MA	13.21 (7.85,18.58)[1)]	6.90 (4.56,9.24)[1)]	2.40 (0.62,4.19)	0			
MA	18.81 (13.51,24.11)[1)]	12.50 (10.30,14.69)[1)]	8.00 (6.41,9.59)	5.60 (4.78,6.41)[1)]	0		
CA	19.11 (13.54,24.68)[1)]	12.80 (10.02,15.57)[1)]	8.30 (6.58,10.02)[1)]	5.90 (4.01,7.78)[1)]	0.30 (-1.40,2.00)	0	
WN	21.74 (15.56,27.93)[1)]	15.43 (11.56,19.31)[1)]	10.93 (7.37,14.50)[1)]	8.53 (5.44,11.62)[1)]	2.93 (-0.26,6.13)	2.63 (-0.98,6.25)	0

Note:SN=silver needle；NK=needle knife；CA=common acupuncture；WN=warming needle；MA=massage

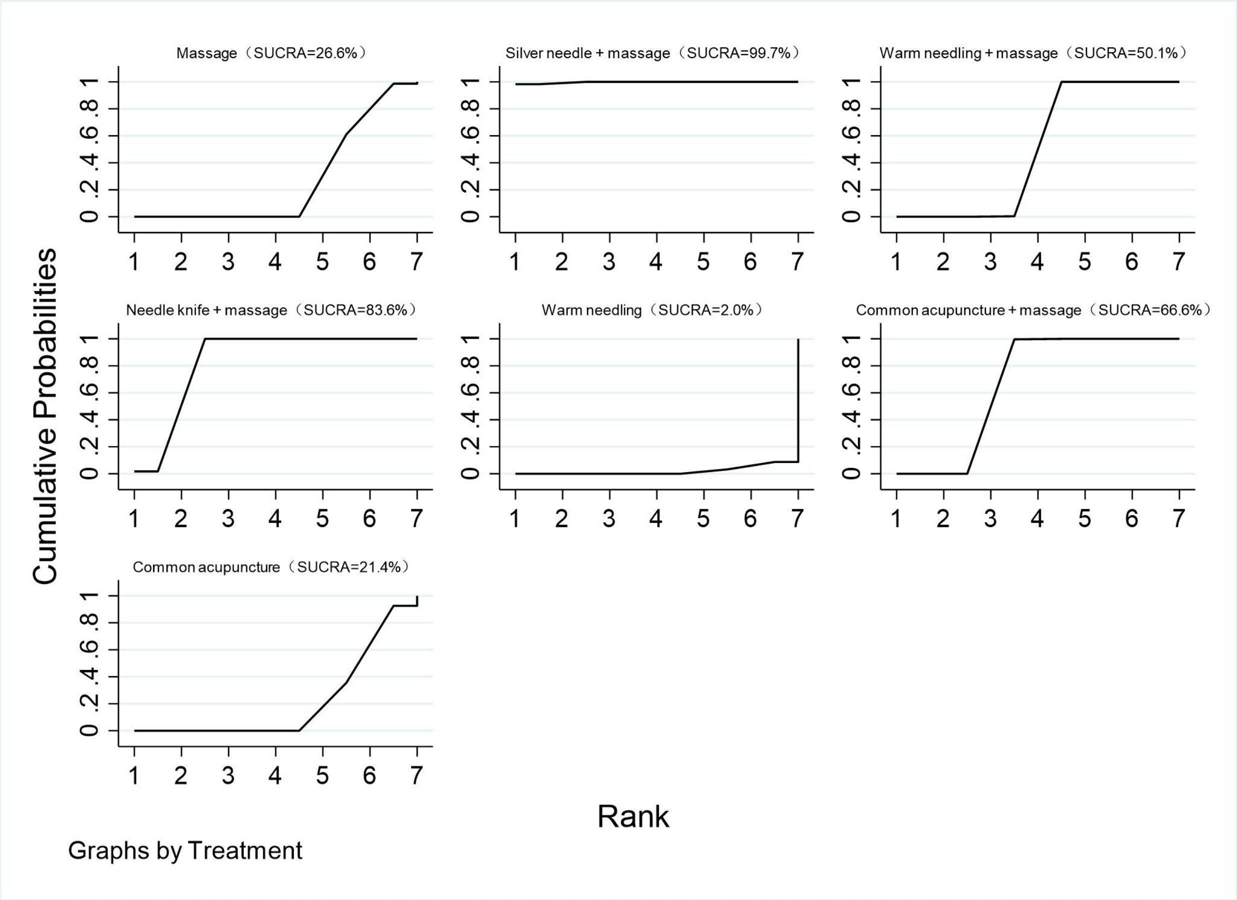

Figure S26 Sucra of Total lysholm score(<3weeks group)

Total Lysholm Score(⩾3weeks group)

Table S16 Network Meta-analysis of Total lysholm score(⩾3weeks group)

Treatment	EA+MA	NK+MA	WN+MA	MA	CA+MA	EA	CA
EA+MA	0						
NK+MA	1.46 (-2.38,5.30)	0					
WN+MA	4.67 (-1.65,10.99)	3.21 (-1.82,8.24)	0				
MA	11.19 (3.87,18.50)[1)]	9.73 (3.49,15.97)[1)]	6.51 (2.98,10.05)[1)]	0			
CA+MA	11.63 (6.33,16.93)	10.17 (6.51,13.83)[1)]	6.96 (3.45,10.47)[1)]	0.44 (-4.76,5.64)	0		
EA	14.00 (11.17,16.83)[1)]	12.54 (9.94,15.14)[1)]	9.33 (3.67,14.98)[1)]	2.81 (-3.94,9.57)	2.37 (-2.12,6.86)	0	
CA	22.84 (15.63,30.05)[1)]	21.38 (15.28,27.48)[1)]	18.17 (12.15,24.18)[1)]	11.65 (4.52,18.79)[1)]	11.21 (6.33,16.09)[1)]	8.84 (2.21,15.47)[1)]	0

Note:EA=electroacupuncture；NK=needle knife；WN=warming needle；CA=common acupuncture；MA=massage

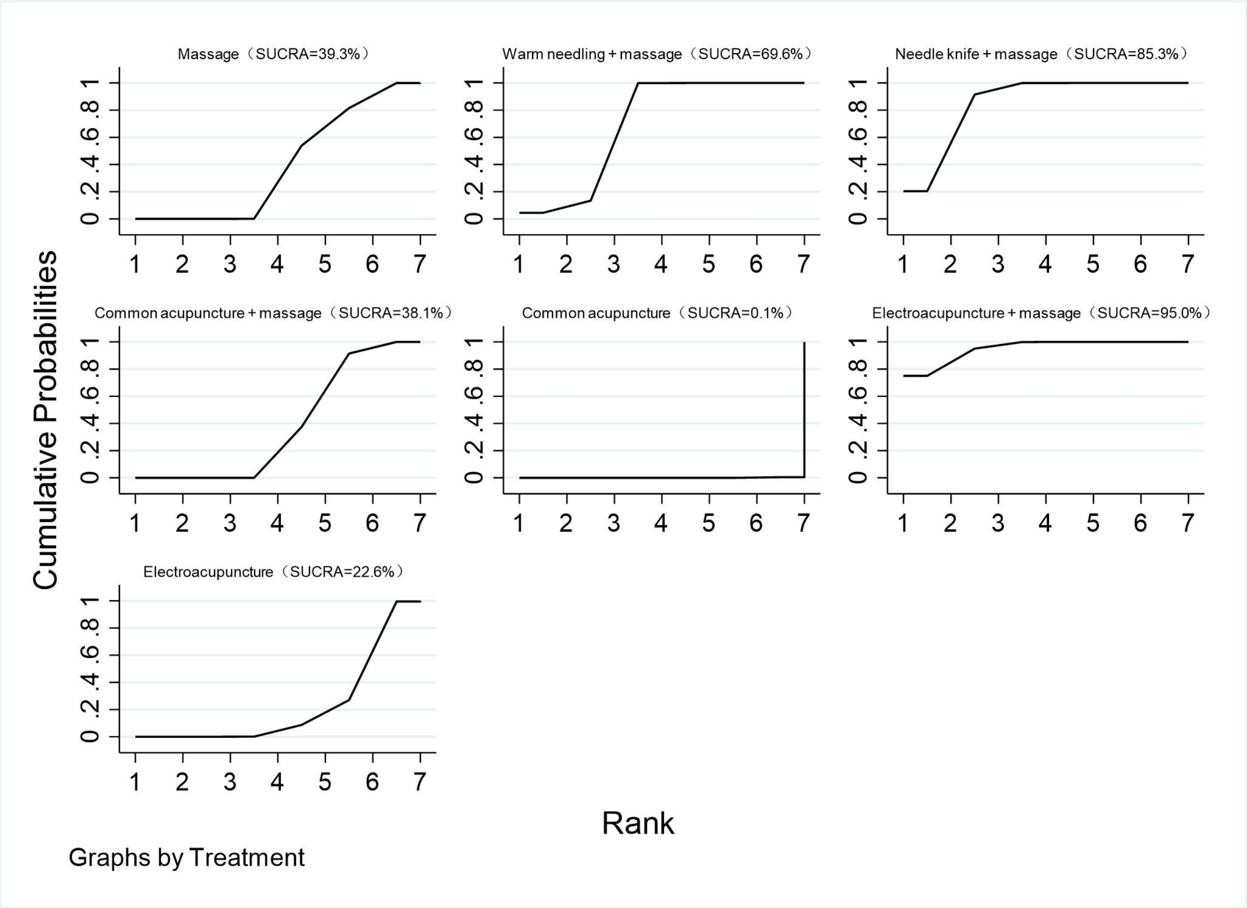

Figure S27 Sucra of Total lysholm score(≥3weeks group)

Table S17 Introduction to different acupuncture and massage therapies

Electrothermal needle： it is a new acupuncture treatment method invented according to the principle of electric needle, which can make the needle body heat up and treat diseases after penetrating into the acupoint of the body. **Operation:** select the acupoint or stimulation site, disinfect the needle quickly into the skin, and then slowly and slightly twist the needle to reach the due depth, find the needle sense, enhance the needle sense to the required degree. Switch on the controller, turn on the heating button and slowly make the pointer of the instrument rise. Stop when the required temperature is reached.

Floating needle： Floating needle therapy is a kind of acupuncture therapy that uses disposable floating needles and other needles to clean the superficial subcutaneous fascia around the local pain. It is a combination of traditional acupuncture and modern medicine. **Operation:** after inserting the needle, take the needle point as the fulcrum, hold the needle holder by hand, swing from side to side, make the needle body fan-shaped movement.

Fire needle:it is a method to treat diseases by stabbing the point quickly with the point of burning red. **Operation:** when needling, use a red-hot needle to quickly Pierce into the selected acupoint, that is, to quickly withdraw the needle.

Silver needle: The silver needle is made of 80% silver and has good heat conduction function. **Operation:** connect the equipment, turn on the heating button, and slowly make the pointer of the instrument rise. Stop when the required temperature is reached.

Warm needle:Warm needle is in the application of acupuncture at the same time to warm stimulation of a therapy. **Operation:** After gas is injected into the needle, mugwort or moxa is placed on the needle handle to make heat pass through the needle into the body.

Beryllium needle:A needle shaped like a sword with blades on both sides. **Operation:** Aim the needle tip at the center of the skin "cross" indentation, and quickly insert the needle. When the needle tip touches the deep fascia, release it.

Electroacupuncture:it is a kind of therapy to stimulate acupoints and treat diseases by inserting acupuncture points to get qi and passing (sensing) the micro current wave of human bioelectricity on the needle. **Operation:** after acupuncture, adjust the output potentiometer to "0", connect the two wires arbitrarily on the two needle handles, then turn on the power switch, select the wave type, and slowly increase the output current to the required.

Needle knife: A needle tool made of metal material that resembles both a needle and a knife in shape. The width of the needle knife is usually equal to the diameter of the needle body and the edge is sharp. **Operation:** After inserting the needle, select a certain technique to release and peel the adhesive tissue fibers.

Catgut embedding needle knife:it is a newly developed instrument based on needleknife, which can be used for embedding at the same time while releasing adhesion. **Operation:** Hold the catgut embedding needle knife, Pierce into the acupoint, bury the thread body into the subcutaneous tissue,

then rotate and exit the catgut embedding needle knife.

Common acupuncture： it is a kind of needle made of stainless steel. **Operation:** After penetrating the skin, the most common method is to lift, insert, twist and other techniques to achieve the purpose of gas.

Note: electrothermal needle, silver needle and warm needle are all inserted into the skin and then connected to the device or inserted into mugwort and mogwort to achieve the purpose of ditherability. The materials of the three are different. Needle knife, buried needle knife and beryllium needle all have blades with different shapes and operating methods.

Chinese traditional tuina： The tuina method is carried out under the guidance of the "Theory and Practice of Tuina", and there are certain regulations on the operation steps, including pushing, take, mention, knead, point, pull, etc.

Joint mobilization： Joint mobilization technology is a highly targeted manual manipulation technique performed by the therapist within the movable range of joint motion. It belongs to the category of passive motion, and its operation speed is slower than that of massage. In application, the physiological motion and accessory motion of the joint are often selected.The main operations include swing, roll, slide, and rotate.

Table S18 The checklist of the PRISMA for network meta-analysis

Section/Topic	Item #	Checklist Item	Reported on Page #
**TITLE**			
Title	1	Identify the report as a systematic review *incorporating a network meta-analysis (or related form of meta-analysis).*	**1**
**ABSTRACT**			
Structured summary	2	Provide a structured summary including, as applicable:<br>**Background:** main objectives<br>**Methods:** data sources; study eligibility criteria, participants, and interventions; study appraisal; and *synthesis methods, such as network meta-analysis.*<br>**Results:** number of studies and participants identified; summary estimates with corresponding confidence/credible intervals; *treatment rankings may also be discussed. Authors may choose to summarize pairwise comparisons against a chosen treatment included in their analyses for brevity.*<br>**Discussion/Conclusions:** limitations; conclusions and implications of findings.<br>**Other:** primary source of funding; systematic review registration number with registry name.	**1-2**
**INTRODUCTION**			
Rationale	3	Describe the rationale for the review in the context of what is already known*, including mention of why a network meta-analysis has been conducted.*_	**2-3**
Objectives	4	Provide an explicit statement of questions being addressed, with reference to participants, interventions, comparisons, outcomes, and study design (PICOS).	**3-4**
**METHODS**			
Protocol and registration	5	Indicate whether a review protocol exists and if and where it can be accessed (e.g., Web address); and, if available, provide registration information, including registration number.	**1**
Eligibility criteria	6	Specify study characteristics (e.g., PICOS, length of follow-up) and report characteristics (e.g., years	

		considered, language, publication status) used as criteria for eligibility, giving rationale. *Clearly describe eligible treatments included in the treatment network, and note whether any have been clustered or merged into the same node (with justification).*_	**4-5**
Information sources	7	Describe all information sources (e.g., databases with dates of coverage, contact with study authors to identify additional studies) in the search and date last searched.	**4-6**
Search	8	Present full electronic search strategy for at least one database, including any limits used, such that it could be repeated.	**SM2-3**
Study selection	9	State the process for selecting studies (i.e., screening, eligibility, included in systematic review, and, if applicable, included in the meta-analysis).	**5**
Data collection process	10	Describe method of data extraction from reports (e.g., piloted forms, independently, in duplicate) and any processes for obtaining and confirming data from investigators.	**4-5**
Data items	11	List and define all variables for which data were sought (e.g., PICOS, funding sources) and any assumptions and simplifications made.	**4-5**
**Geometry of the network**	**S1**	Describe methods used to explore the geometry of the treatment network under study and potential biases related to it. This should include how the evidence base has been graphically summarized for presentation, and what characteristics were compiled and used to describe the evidence base to readers.	
Risk of bias within individual studies	12	Describe methods used for assessing risk of bias of individual studies (including specification of whether this was done at the study or outcome level), and how this information is to be used in any data synthesis.	**11**
Summary measures	13	State the principal summary measures (e.g., risk ratio, difference in means). *Also describe the use of additional summary measures assessed, such as treatment rankings and surface under the cumulative ranking curve (SUCRA) values, as well as modified approaches used to present summary findings from meta-analyses.*	**5-6**
Planned methods of analysis	14	Describe the methods of handling data and combining results of studies for each network meta-analysis. This should include, but not be	**5-6**

		limited to:<br>• *Handling of multi-arm trials;*<br>• *Selection of variance structure;*<br>• *Selection of prior distributions in Bayesian analyses; and*<br>• *Assessment of model fit.*	
**Assessment of Inconsistency**	**S2**	Describe the statistical methods used to evaluate the agreement of direct and indirect evidence in the treatment network(s) studied. Describe efforts taken to address its presence when found.	**5-6**
Risk of bias across studies	15	Specify any assessment of risk of bias that may affect the cumulative evidence (e.g., publication bias, selective reporting within studies).	**11**
Additional analyses	16	Describe methods of additional analyses if done, indicating which were pre-specified. This may include, but not be limited to, the following:<br>• Sensitivity or subgroup analyses;<br>• Meta-regression analyses;<br>• *Alternative formulations of the treatment network; and*<br>• *Use of alternative prior distributions for Bayesian analyses (if applicable).*_	**26-28**
**RESULTS†**			
Study selection	17	Give numbers of studies screened, assessed for eligibility, and included in the review, with reasons for exclusions at each stage, ideally with a flow diagram.	**6-7**
**Presentation of network structure**	**S3**	Provide a network graph of the included studies to enable visualization of the geometry of the treatment network.	**13-22**
**Summary of network geometry**	**S4**	Provide a brief overview of characteristics of the treatment network. This may include commentary on the abundance of trials and randomized patients for the different interventions and pairwise comparisons in the network, gaps of evidence in the treatment network, and potential biases reflected by the network structure.	**13-26**
Study	18	For each study, present characteristics for which	**6-10**

characteristics		data were extracted (e.g., study size, PICOS, follow-up period) and provide the citations.	
Risk of bias within studies	19	Present data on risk of bias of each study and, if available, any outcome level assessment.	**11**
Results of individual studies	20	For all outcomes considered (benefits or harms), present, for each study: 1) simple summary data for each intervention group, and 2) effect estimates and confidence intervals. *Modified approaches may be needed to deal with information from larger networks.*	**13-26**
Synthesis of results	21	Present results of each meta-analysis done, including confidence/credible intervals. *In larger networks, authors may focus on comparisons versus a particular comparator (e.g. placebo or standard care), with full findings presented in an appendix. League tables and forest plots may be considered to summarize pairwise comparisons.* If additional summary measures were explored (such as treatment rankings), these should also be presented.	**13-26**
**Exploration for inconsistency**	**S5**	Describe results from investigations of inconsistency. This may include such information as measures of model fit to compare consistency and inconsistency models, *P* values from statistical tests, or summary of inconsistency estimates from different parts of the treatment network.	**13-26**
Risk of bias across studies	22	Present results of any assessment of risk of bias across studies for the evidence base being studied.	
Results of additional analyses	23	Give results of additional analyses, if done (e.g., sensitivity or subgroup analyses, meta-regression analyses*, alternative network geometries studied, alternative choice of prior distributions for Bayesian analyses,* and so forth).	**SM12-27**
**DISCUSSION**			
Summary of evidence	24	Summarize the main findings, including the strength of evidence for each main outcome; consider their relevance to key groups (e.g., healthcare providers, users, and policy-makers).	**28-30**
Limitations	25	Discuss limitations at study and outcome level (e.g., risk of bias), and at review level (e.g., incomplete retrieval of identified research, reporting bias). *Comment on the validity of the assumptions, such as transitivity and consistency. Comment on any concerns regarding network geometry (e.g.,*	**31**

		avoidance of certain comparisons).	
Conclusions	26	Provide a general interpretation of the results in the context of other evidence, and implications for future research.	**31**
**FUNDING**			
Funding	27	Describe sources of funding for the systematic review and other support (e.g., supply of data); role of funders for the systematic review. This should also include information regarding whether funding has been received from manufacturers of treatments in the network and/or whether some of the authors are content experts with professional conflicts of interest that could affect use of treatments in the network.	**1**

PICOS = population, intervention, comparators, outcomes, study design.

* Text in italics indicateS wording specific to reporting of network meta-analyses that has been added to guidance from the PRISMA statement.

† Authors may wish to plan for use of appendices to present all relevant information in full detail for items in this section.
